# Advancing the Performance of Anion Exchange Membrane Electrolysis by Employing a Powder-Based Ionomer during Anode Catalyst Layer Fabrication

**DOI:** 10.1021/acsaem.5c03719

**Published:** 2026-02-19

**Authors:** Ai-Lin Chan, Arielle L. Clauser, Makenzie R. Parimuha, James L. Young, Joshua D. Sugar, Shaun M. Alia

**Affiliations:** a Chemical and Material Sciences Center, 53405 National Renewable Energy Laboratory, 15013 Denver West Parkway, Golden, Colorado 80401, United States; b 111651 Sandia National Laboratories, Livermore, California 94550, United States

**Keywords:** anion exchange membrane water electrolysis, electrode fabrication, catalyst/ionomer distribution, powdered form of ionomer, distribution of relaxation times (DRT), impedance modeling

## Abstract

The performance of anion exchange membrane water electrolysis (AEMWE) can be significantly improved by utilizing powdered ionomers during the fabrication of the anode catalyst layer (CL) to modify the CL properties. When comparing powdered ionomers to dispersed ionomers across various catalysts—including cobalt oxide (Co_3_O_4_), nickel–iron oxide (NiFe_2_O_4_), and iridium oxide (IrO_2_)—the anode fabricated with powdered ionomers demonstrates improved performance in polarization curves, enhanced charge transfer kinetics, and reduced ohmic and transport losses, as evidenced by voltage breakdown and electrochemical impedance spectroscopy analyses. Optimal performance is achieved using a Co_3_O_4_ catalyst with a 10 wt % powdered ionomer via the catalyst-coated substrate method. Microscopy analyses reveal that electrodes formed with powdered ionomers during fabrication exhibit a more uniform catalyst and ionomer distribution, increased porosity with smaller pore areas, improved electronic conduction with less catalyst agglomeration isolated by a nonconductive ionomer, and enhanced interfacial contact with the membrane and transport layer. These findings highlight that ionomers in a powdered form can promote beneficial properties and are a promising approach to improving AEMWE efficiency.

## Graphical abstract

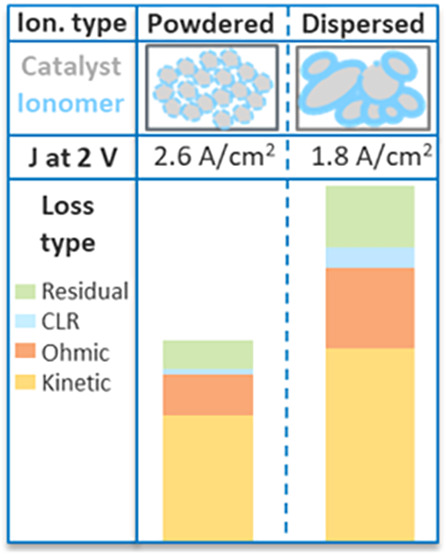

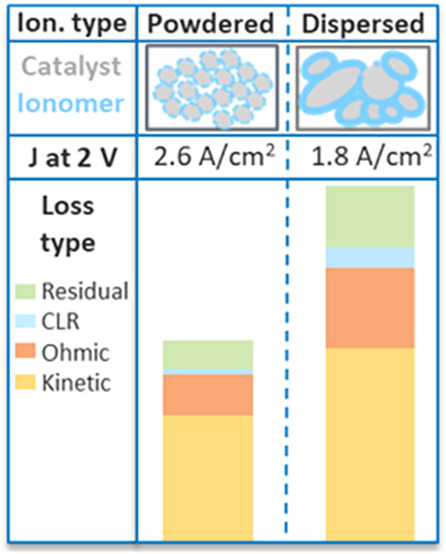

## 1 Introduction

Hydrogen demand has gradually increased for various end-use applications, such as refining processes in transportation, chemical production in agriculture, steel manufacturing, and grid support.^1,2^ Further hydrogen market expansion, however, requires addressing affordability concerns. Water splitting technologies are promising, particularly when coupled with low-cost and intermittent power sources.^2,3^ Low-temperature electrolysis has been commercially available for liquid alkaline water electrolysis (LAWE) and proton exchange membrane water electrolysis (PEMWE) and provided large-scale (multimegawatt) hydrogen production. Traditional LAWE systems face limitations such as lower operating current and challenges with load-following, primarily due to the electrode–separator distance. Additionally, operating costs are higher because of the use of a concentrated caustic electrolyte (7 M KOH) and the need for downstream hydrogen compression.^4^ The limitations of PEMWE further include lifetime challenges when thrifting platinum group metal (PGM) content to reduce capital cost and the scarcity of PGM components.^2,3,5^ Anion exchange membrane water electrolysis (AEMWE) seeks to integrate the advantages of LAWE and PEMWE, such as the use of PGM-free components with enhanced durability in high-pH environments, greater efficiency under differential pressure, and reduced operating costs due to lower electrolyte concentration, driving interest in emerging research opportunities.^3,5^

Within AEMWE research, catalyst screening and development with abundant non-PGM metals (Co, Ni, Cu, and Fe) have focused on enhancing oxygen evolution reaction (OER) kinetics at the anode by improving activity, electronic conductivity, and stability in an alkaline electrolyte.^6–9^ Platinum (Pt)-based catalysts are widely used at the cathode in AEMWE due to the lower hydrogen evolution reaction (HER) overpotential. Anion exchange polymers are engineered to bind and disperse catalyst particles while ensuring high ionic conductivity for OH^–^ transport within the electrodes and the anion exchange membrane (AEM); however, optimizing the balance between ion exchange capacity and water uptake is necessary to enhance membrane robustness.^10–12^ Electrode performance can be enhanced through catalyst layer (CL) optimization, which involves adjusting the catalyst–ionomer–pore composition and distribution, refining the size of catalyst particles and agglomerates, and tailoring the hydrophobic properties of the electrode.^13–16^ Ni et al. examined the distribution of primary and secondary pores within the cathode CL and evaluated its impact on proton exchange membrane fuel cell performance, particularly focusing on liquid saturation and gas diffusion.^17^ In AEM fuel cells, ethylene-*co*-tetrafluoroethylene (ETFE)-based radiation-grafted powders were fabricated as anion exchange ionomers and applied in the CL in several studies.^18–21^ The ionomer particle size needs to be optimized with different catalysts to have a better distribution of the catalyst/ionomer and improved CL morphology. He et al. also applied powdered ionomers with a sulfonamide-linked alkyl ammonium perfluorinated anion exchange polymer and confirmed that powdered samples have higher peak power densities compared to dispersed samples when tested with different cathode catalysts.^22^ The potential benefits of using a powdered form of ionomers included reducing kinetic, ohmic, and mass transport losses in fuel cells.^22^ A CL with higher porosity may further enhance water management by maintaining hydration while minimizing electrode flooding and drying out. Despite these advantages in fuel cells, the comparison between powdered and dispersed ionomer forms has not been reported in AEMWE, which poses significant interest in understanding catalyst–ionomer interactions to improve AEMWE cell efficiency and ensure long-term operational stability.

This work aims to highlight the performance improvements achievable in AEMWE by implementing powdered ionomers in the anode CL. Anodes fabricated using the catalyst-coated substrate (CCS) method showed higher efficiency compared to three other catalyst-coated membrane (CCM) approaches due to a combination of factors. With the evaluation of three different catalysts—cobalt oxide, Co_3_O_4_, nickel–iron oxide, NiFe_2_O_4_, and iridium oxide, IrO_2_—and two ionomer types, powdered-fabricated electrodes also consistently delivered higher performance. Voltage breakdown analysis, electrochemical impedance spectroscopy, and microscopy provide mechanistic explanations for the improved polarization behavior of powdered ionomer systems observed in the study. These findings demonstrate the need for understanding how the catalyst, ionomer, and pore distribution impact AEM electrolyzer performance and the potential improvements possible by modifying the electrode structure.

## 2 Experimental Section

### 2.1 Membranes and Ionomers

Anion exchange membranes and ionomers with two different chemical backbones were tested in this work. A PiperION-A TP-85 (Versogen, 80 μm) membrane and its ionomers (powdered and dispersed forms with 5 wt % in ethanol) were based on poly­(aryl piperidinium) polymers. An alkyl ammonium perfluorinated membrane (PF AEM, Gen2, 60 μm) and the ionomer were prepared in a previous study.^23^ The dispersion form of Gen2 was prepared by dissolving PF AEM powder in a 5–10 wt % dimethylacetamide (DMAc) solution.

### 2.2 Electrode Fabrication

Carbon-supported Pt (TEC10E50E from Tanaka Kikinzoku Kogyo) as the cathode catalyst was added to deionized water (DIW) and *n*-propyl alcohol (Sigma, OmniSolv, ≥99.5%). The ratio of DIW and *n*-propyl alcohol was 5.7 to 4.3. The ink was sonicated in an ice bath for 5 min, and then, 20 wt % of dispersed ionomer from Versogen or Gen2 was added to the ink, followed by a series of sonication procedures (30 s horn, 20 min bath, and 30 s horn). During the horn sonication (Qsonica Ultrasonic Sonicator, S-4000, 0.5 in. horn), the ink bottle was placed in a beaker filled with ice and the intensity for sonication was set as 1% amplitude. The ink was sprayed on a 5 cm^2^ carbon gas diffusion layer (GDL from Fuel Cell Technologies, MGL280) with an airbrush gun (Grex Tritium TG3, 0.3 mm nozzle) on a vacuum plate at 80 °C.

Non-PGM and PGM catalysts were used for anode fabrication: Co_3_O_4_ (US Research Nanomaterials, Inc., 99%), NiFe_2_O_4_ (US Research Nanomaterials, Inc., 98%), and IrO_*x*_ (Alfa Aesar, 43396, 99%). The fabrication process of powder-based anodes was adjusted from the previous works.^12,20^ The powdered ionomer (1–30 wt %, ionomer to catalyst ratio) was ground with an agate mortar and pestle for 5 min to reduce agglomerate size, and then, the catalysts were mixed with the ionomer and ground for another 5 min. DIW (10% of total ink volume) was added and mixed in the mortar, after which *n*-propyl alcohol (90% of total ink volume) was added, ground, and transferred to a vial three times. The ink was then horn-sonicated for 10 s and bath-sonicated for 30 min before spraying. The anode ink with the dispersed ionomer (20 wt %) was prepared with the same processes as the cathode ink, while the ratio of DIW to *n*-propyl alcohol was 1 to 9 and the catalyst content was the same to keep the consistency between powdered and dispersed samples. The powdered and dispersed inks of non-PGM catalysts and the Versogen ionomer were evaluated at 25 °C with dynamic light scattering (DLS, Malvern Instruments Ltd., Malvern). The composition of water and alcohol for DLS measurement was the same as for MEA testing with 20 wt % of ionomer, while the catalyst was 0.006 wt % of the total ink for the quality of DLS results. DLS was implemented with at least 5 runs for each sample. Each run contained 13–15 measurements, depending on the data quality. The reported DLS results were from the first and fourth runs to include ink stability and the agglomeration process of different forms of ionomers.

The dispersed ionomer contained both ethanol and *N*,*N*-dimethylacetamide (DMAc), which was not matrix-matched in the powdered inks due to the unknown and proprietary composition of the ionomer dispersion. While ethanol has a boiling point (78 °C), dipole moment (1.69 D), and dielectric constant (24.5 at 25 °C) similar to those of *n*-propyl alcohol (82.3 °C, 1.6 D, and 20 at 25 °C, respectively), DMAc is dissimilar (165 °C, 3.72 D, and 37.8 at 25 °C, respectively) and may affect ink wettability and drying. The small volume of ionomer dispersion within the ink (211 μL of ionomer dispersion into 4 mL of ink) and the small DMAc within the ionomer dispersion may limit the impact on the ink properties. The user-controlled aspect of airbrushing further ensured complete ink drying between spray passes and confirmed minimal differences in ink drying, albeit visual.

Differences in the ink composition, mixing, and grinding procedures and the user-controlled aspect of airbrushing may affect ink rheology and the resulting catalyst layer properties and cell efficiencies. To help understand the impact of the ionomer form and airbrushing procedures on the ink rheology, DLS measurements were completed on powdered and dispersed inks for the non-PGM catalysts. Additionally, the XRF catalyst loadings (0.75 ± 0.11 mg_Metal_/cm^2^) demonstrated reproducibility and consistency in the airbrushing of catalyst layers with ionomers in both powdered and dispersed forms.

The CCS method and three CCM methods were applied to create a 5 cm^2^ anode CL (Table 1). For the CCS, the procedure was the same as during the cathode fabrication, by spraying inks onto Ni porous transport layers (PTLs from Bekaert, BEKIPOR 2NI 18-0.25) with an airbrush gun onto a vacuum plate at 80 °C. CCM1 and CCM2 were coated with an airbrush gun and a paint brush onto PiperION-A TP-85 membranes, respectively, fixed onto a vacuum plate at 80 °C. For CCM3, the ink was first sprayed on an ethylene tetrafluoroethylene (ETFE) film, which was then soaked in 1 M NaCl (Sigma-Aldrich, BioXtra, ≥98%) for an hour (refreshed solution twice) to ion exchange in Cl^–^ forms before being decal-transferred onto the membrane at 140 °C and 140 kg/cm^2^ for 40 min.^18^

**Table 1:**
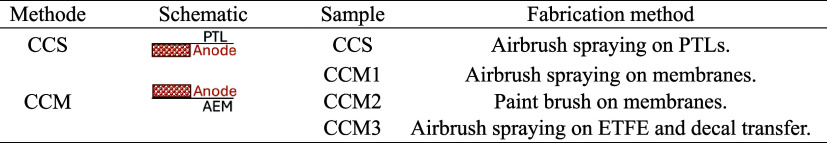
Summary of Anode Fabrication by Different Methods

After the ink deposition steps, the catalyst loadings were checked with an X-ray fluorescence spectrometer (XRF; Fisher XDV-SDD XRF). At least three different spots in the active area were measured, and the average catalyst loadings were calculated with standard deviation. The cathode loading was 0.18 ± 0.02 mg_Pt_/cm^2^. Differences in the anode loading were found and can alter cell performance including site-access, kinetics, and electron transport (CLR and HFR). The anode loadings reported here (0.5–1 mg_Metal_/cm^2^), however, were previously found to result in similar catalyst utilization and cell performances and may be related to site-access limitations due to suboptimal CL properties (catalyst, ionomer, and pore distribution) that may minimize utilization differences between catalyst loadings with comparatively small differences and contributions from the anode PTL minimizing smaller differences in catalyst loading when operating in a supporting electrolyte.^14^

### 2.3 MEA Testing

Before testing, PiperION-A TP-85 membranes were soaked in 3 M KOH (Sigma-Aldrich, pellets for analysis EMSURE, ≥85%) for 48 h and the KOH solution was refreshed at the end of the first day to ion exchange the membrane from the carbonate form to the hydroxide form. Catalyst-coated PTLs and GDLs were soaked in the same KOH solution for at least 20 min before cell assembly. An alkyl ammonium perfluorinated membrane (Gen2) and the electrodes were ion exchanged for 24 and 1 h, respectively, prior to testing. The cell hardware consisted of stainless-steel end plates (316L), Au-coated current collectors, and Ni-based flow fields. In the cell assembly for both CCS and CCM, PTFE (polytetrafluoroethylene) gaskets with the thickness of 280 μm were used at both electrodes to have around 20% compression with a 4.5 N m torque.

The tests were performed at 80 °C, with 50 mL/min of 1 M KOH supplied to the cathode and anode. MEA testing was completed with a potentiostat (Autolab PGSTAT302N) and a 20 A booster (Metrohm Autolab). The break-in procedure included 2 V for 2 h at 80 °C, followed by polarization curves, which were taken from 1.4–2.0 V, with 120 s hold per voltage. The presented polarization curves were from current responses to the applied voltages. Electrochemical impedance spectroscopy (EIS) was then measured in a potentiostatic mode at the same voltages as in the polarization curves, with sinusoidal waves from 18k to 0.1 Hz, with 0.6 mV perturbation and 10 points collected per decade. In the voltage breakdown analysis, the high frequency resistance (HFR) extracted from EIS measurements was used to generate HFR-free voltages (*V*_HFR‑free_) for Tafel analysis. In situ catalyst layer resistance (CLR) was obtained by nonfaradaic impedance (NFI) measured at 1.25 V, with the linear approximation method developed in a previous study.^24^ While ideal blocking conditions are not fully realized in AEM systems, the linear extrapolation performed in this study was limited to a specific frequency range and repeated across various samples. Given that the cathode materials used were identical, NFI was employed as a comparative and consistency-driven method to analyze trends associated with different anode configurations. The polarization curves were then broken down into kinetic, ohmic, CLR, and other contributions. Cyclic voltammetry (CV) was taken in the range of 0–1.4 V at three scan rates (100, 50, and 20 mV/s) followed by EIS.

### 2.4 Ex Situ Characterization

Samples were prepared for electron microscopy and energy-dispersive X-ray spectroscopy by cutting small sections of the electrode, which had undergone an ion exchange process to exchange OH^–^ to I using 5.0 M KI and adhering them to 0.25 in SEM stubs using carbon tape. The samples were then carbon-coated before being imaged using an FEI Helios NanoLab 600i DualBeam SEM/FIB to collect top-down images and using an Everhart–Thornley detector (ETD) to collect secondary electron images. EDS maps were collected at 15 kV and 6.4 nA using Oxford’s AZtec software and Oxford 170 mm2 Ultim Max SDD EDS detector. Focused ion beam cross sections were made of samples in representative areas, and the corresponding EDS maps were collected with the same parameters as in the top-down images. To calculate the fraction of porosity and ionomer in the CL at the PTL interface, cross-sectional SEM images were prepared using a Helios Nanolab 660 FIB/SEM. A specific region was selected for sectioning, and a trench was created in front of the region using the FIB. The cross-sectional face was then polished with progressively smaller ion currents at 30 kV, followed by a final Ga ion beam polish of 2.5 nA. SEM images were acquired at an accelerating voltage of 2 or 5 kV and a 52° viewing angle. The representative images were segmented into distinct components—porosity, ionomer, catalyst, PTL, and FIB Pt—using the image classification routines in the software ilastik.^25^ The segmentation process produced individual layers corresponding to each component. These layers were subsequently processed using custom Python scripts to calculate the connected area and area fraction of the various components.

Samples for measuring ex situ the CL in-plane sheet resistance (*R*_sheet_) were made by hand-spraying catalyst ink onto Nafion 115 membranes with a final loading of ∼0.6 mg/cm^2^. The final CL area is an average of 23.5 × 23.5 mm^2^. Using a colinear four-point probe, CV was performed from −10 to 10 mV versus the open circuit potential and the resulting polarization curve fit by linear regression. The inverse slope is raw measured resistance *r*, which is converted to *R*_sheet_ using the geometrically derived relationship, *R*_sheet_ = 4.53*r*. Values are reported in Ω/square.^26^

Samples for measuring ex situ CL through-plane resistance were made by hand-spraying catalyst ink onto Ni-PTLs with a final loading of ∼0.6 mg/cm^2^. These porous transport electrodes (PTEs) were then cut to 5 cm^2^. Interfacial contact resistance (ICR) measurements were performed by placing the PTEs between two pieces of carbon GDL (Sigracet 29BC) with the microporous layer facing the PTE.^27^ Two gold-coated copper plates were used to compress the GDL/PTE/GDL stack while applying a constant 0.5 A/cm^2^ current density and increasing the applied force incrementally, from 50 to 550 N, and the voltage response is recorded. The values of voltage drop were taken at 550 N and divided by current density, and thus, the area-specific ICR is reported in Ω cm^2^.

### 2.5 Impedance Modeling

The EIS results were analyzed by the Kramers–Kronig (KK) test, equivalent circuit model (ECM), and distribution of relaxation time (DRT) methods to further probe electrochemical processes with different characteristic frequencies. The KK test and ECM were performed with a Python package.^28^ The DRT analysis was implemented with an open-source MATLAB package (DRTools), developed by Ciucci et al.^29,30^ The validation and the linearity of impedance data were examined with KK relations, and an example of the KK test is shown in Figure S1. The modeled real and imaginary impedance values with residual errors >1% were removed from the impedance data before ECM and DRT analysis. The equivalent circuit used in this study is illustrated in Figure S2, with an ohmic resistance (or high frequency resistance, HFR) in series with two time constants (charge transfer resistances (*R*_ct,1_ and *R*_ct,2_) in parallel with constant phase elements (CPE1 and CPE2). The DRT input parameters are listed in Figure S3. A Gaussian discretization method and the first order of a regularization derivative were applied. The regularization parameter (λ) was selected as 1 × 10^–3^ based on the sensitivity analysis (Figure S3). The DRT peak deconvolution and analysis were studied in a previous publication.^31^ An example of DRT results at different operating voltages is shown in Figure S4, which confirmed a kinetic process indicated by the exponential decrease with increasing voltage.

## 3 Results and Discussion

### 3.1 Electrode Deposition Methods

Different electrode fabrication methods were applied with Co_3_O_4_ as the anode catalyst using ionomers as-supplied in a powdered and dispersed form. While the ionomer form contributes to electrode and cell differences, the form also impacts ink rheology and allowed for differences in ink chemistry, solvent and ink additives, and dispersion processing histories that may have an effect as well. Throughout this study, electrodes fabricated using the as-supplied ionomers are referred to as powdered and dispersed for naming simplicity and are not intended to oversimplify factors affecting the electrode fabrication process or their differences.

Figure 1 presents the results for dispersed and powdered forms of the ionomer. For the dispersed ionomer anode in Figure 1a, the CCS outperformed the three CCM methods (CCM1: airbrush spray; CCM2: paint brush; CCM3: decal transfer), with a higher current response at 2 V (CCS: 2.32 A/cm^2^, CCM: 1.55–1.62 A/cm^2^). In the voltage breakdown analysis at 1 A/cm^2^ in Figure 1c, the performance differences were mainly due to other (mass transport), ohmic, and CLR losses, while the kinetic contributions were similar (0.51–0.52 V). CCM3 (decal transfer) had a slightly lower kinetic overpotential than CCS (7 mV), and EIS at 1.5 V showed a similar *R*_ct_ (Figure S5). The improved OER kinetics obtained by CCM3 (decal transfer) may be because the CL had better contact to the membrane that improved site-access.^32^ The difference in other losses (the overpotentials excluding kinetic, ohmic, and CLR) was pronounced, which might be related to mass transport and generated greater variation between CCS and CCM at higher voltages, as shown in Figure 1a. The CCS method, which deposited the CL at the membrane interface and partially into the PTL pores, may have improved mass transport through catalyst layer segmentation, allowing for improved bubble removal at a macroscopic scale. Although CCM electrode fabrication methods have been reported to improve CL adhesion to the membrane, reducing CL delamination and improving AEMWE stability,^33–35^ variations in the catalyst type and loading could produce different outcomes. During the fabrication processes, applying dispersed ink via spraying (CCM1) or painting (CCM2) on the membranes posed drying challenges and caused ink pooling, potentially reducing anode porosity and exacerbating mass transport limitations. CCM3 (decal transfer) fabrication, involving additional heat and pressure, might have deformed the CL, resulting in a more compact anode that restricted electrolyte and gas transport. Regarding ohmic losses, CCM3 (decal transfer) exhibited a higher HFR compared to other methods, possibly due to membrane damage caused by the decal transfer process, which may have decreased AEM ionic conductivity. The CCS had lower ohmic overpotentials because the dispersed ink, sprayed onto the PTL rather than onto a membrane, allowed deeper penetration into the PTL fibers. This resulted in an increased CL area and enhanced electronic conductivity at the catalyst/PTL interfaces. The CCM methods are expected to have better ionic conduction between the CL and the membrane, which minimize ionic contributions to the CLR;^24,32,36^ however, a lower CLR with the CCS method was observed (CLR approximation in Figure S6), which was likely attributed to differences in interfacial electron transfer rather than ionic conduction, as the system was operated in a concentrated electrolyte (1 M KOH), which minimized ionic resistance.^33^

**Figure 1:**
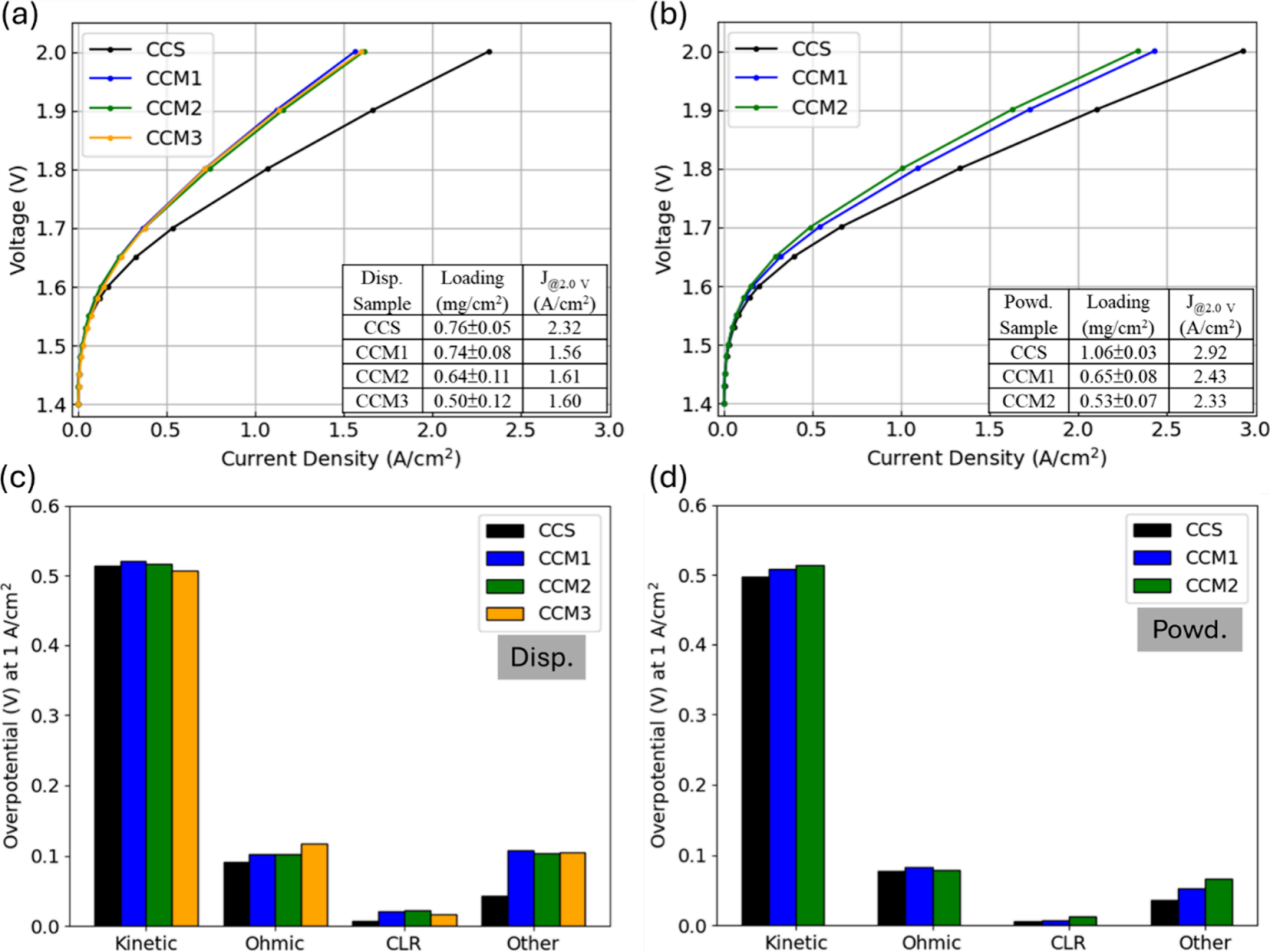
Polarization curves of CCS and CCM methods for Co_3_O_4_ anode catalyst layers with Versogen (a) dispersed (Dips.) and (b) powdered (Powd.) ionomers. (c) and (d) are the voltage breakdown results of (a) and (b) at 1 A/cm^2^, respectively. CCM1, CCM2, and CCM3 denote airbrush spraying, paint brush coating, and decal transfer, respectively.

Similar trends between the CCS and CCM methods were also observed in powder-fabricated samples in Figure 1b,d. These results did not include a decal transfer (CCM3). Multiple attempts to transfer the CL fabricated with powdered ionomers onto the membrane via decal transfer were unsuccessful due to poor adhesion. For the other two CCM methods, the current responses were around 2.33–2.43 A/cm^2^ at 2 V, whereas CCS achieved a higher value (2.93 A/cm^2^), demonstrating better performance compared to the results with the dispersed ionomer in Figure 1a. Further discussion of performance differences between powdered and dispersed samples will be provided in the next section. The CCS performance improvement over the CCMs in Figure 1b resulted from various contributions, including lowering the overpotentials from kinetic (11–17 mV), ohmic (1–6 mV), CLR (1–7 mV), and other (16–30 mV) losses. EIS and CLR results are summarized in Figures S5 and S6. Compared to the CCM methods, higher amounts of active sites with the CCS method were likely achieved by airbrush spraying the catalyst ink into the PTL fibers, leading to better catalyst utilization and improved performance (capacitance from ECM: 0.85 F (CCS); 0.67 F (CCM1); 0.44 F (CCM2)). The performance differences in the ohmic and CLR contributions with powder-fabricated electrodes were not as pronounced as the disperse-based in Figure 1c, possibly due to better distribution of catalyst particles with the powdered ionomer so that the electronic conduction between catalysts was more continuous and had less variation than with the dispersed ionomer. Of note was that the CCM methods were more sensitive to fabrication parameters and produced greater variability in CL structure. When the membrane was flooded with ink during spraying, it deformed and created an uneven surface that can lead to nonuniform catalyst deposition. During airbrush spraying and paint brush coating, the catalyst ink may penetrate the membrane micropores and create mechanical changes. The swelling properties of the membrane can also distort the polymer and lead to poor contact during cell assembly that can create differences in electron/ion transport and result in differences in HFR and CLR. The CCM fabrication methods used in this study were more vulnerable to shorting, leading to cell failures during longer operation times (up to 12 h). Regardless of whether the ionomer was utilized in its dispersed or powdered form, the CCS approach consistently demonstrated a superior performance. Consequently, the CCS methodology provided a more stable platform and was employed in subsequent sections.

### 3.2 Powdered and Dispersed Ionomers

#### 3.2.1 Non-PGM Catalysts

The polarization curves in Figure 2a,b indicate a notable enhancement in performance with the use of anode CLs fabricated with a powdered ionomer. The current responses with a powdered ionomer at 2 V were 46.3% (Versogen) and 20.8% (Gen2) higher for Co_3_O_4_ and 17.8% (Versogen) and 16% (Gen2) higher for NiFe_2_O_4_. In voltage breakdown results, the majority of the samples displayed kinetic overpotentials as the primary contributors to these differences. The slope and intercept of linear fitting between *V*_HFR‑free_ and log­(*i*) (Figure S7 and Table S1) were in a good agreement with the results of Co-, Ni-, and Fe-based catalysts in alkaline systems.^7,14^ A similar Tafel slope was expected for the CL with a similar catalyst loading and the same catalyst; however, the samples utilizing powdered ionomers at the anode consistently had lower Tafel slopes than the dispersed group. These results clearly show that different ionomer forms can directly impact ink and electrode properties and kinetic performance, and the lower Tafel slopes were likely due to differences in site utilization and not reactivity. Uniform catalyst–ionomer distribution in the powder-fabricated samples might allow more effective active surface area accessible to reactants, better wettability and gas permeability in the anode, and lower local OH^–^ transport resistance. These effects may have shifted the local overpotential at the active sites, especially in the 10–100 mA/cm^2^ region where Tafel slopes were extracted. Compared to dispersed-based anodes, kinetic losses were reduced (by ≈30 mV with Co_3_O_4_ and by ≈23 mV with NiFe_2_O_4_) by using the powdered form of ionomers for both Versogen and Gen2 in Figure 2c,d. Despite the fact that Versogen and Gen2 ionomers have different chemical backbones and functional groups, the kinetic performance did not show much variation. It has been suggested that the ionomer plays crucial roles in binding catalysts and facilitating mass transport, while it does not necessarily provide ionic conduction or benefit site reactivity.^15^ Further analysis in impedance interpretation and modeling will be discussed to help better understand the polarization processes and identify different kinetic properties among the tested samples.

**Figure 2:**
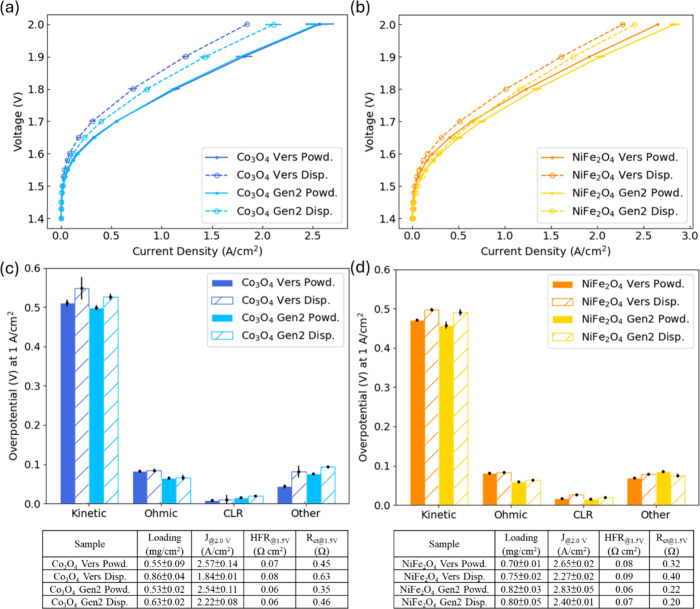
Polarization curves of powdered (Powd.) and dispersed (Disp.) ionomers from Versogen (Vers) and Gen2 with (a) Co_3_O_4_ and (b) NiFe_2_O_4_ in anode catalyst layers. Overpotentials from kinetic, ohmic, CLR, and other losses at 1 A/cm^2^ with (c) Co_3_O_4_ and (d) NiFe_2_O_4_.

For other losses, Co_3_O_4_ with the powdered ionomer outperformed the dispersed by 38 and 19 mV with Versogen and Gen2 ionomers, respectively. Other losses were assumed to be mass transport and water and gas management in the electrodes and transport layers. Figure 3 illustrates that the powder-fabricated anodes had a more porous structure than the dispersed ones, which may have allowed KOH electrolyte and gas transport in more efficient ways and reduced mass transport losses. Postprocessing of representative SEM cross-sectional images (Figure S10a) revealed that the sample utilizing the powdered form exhibited a higher porosity compared to the dispersed. SEM-EDX results in Figure S9 also show that the powder-fabricated anode CL had more homogeneous distribution of catalysts and ionomers, which allowed more uniform transport pathways for ions, KOH, and oxygen. In contrast, the anodes using dispersed ionomers were less porous and may have had more flooding and gas/water management issues, which further covered the active sites between catalyst–ionomer and catalyst–electrolyte interfaces and thus deteriorated OER kinetics.^37^ Compared to the dispersed ionomer, Co_3_O_4_ with the powdered Versogen ionomer was more stable and with a smaller average agglomerate size (109.8 nm versus 131.9 nm from the dispersed) in DLS measurements (Figure S11). The dispersed ionomer resulted in increased agglomeration, as the measurements proceeded. The good agreement between DLS and the representative SEM results further implied that the powdered ionomer helped the ink dispersion during electrode fabrication and improved the site-access within the electrode. For NiFe_2_O_4_, the difference in other losses was less pronounced compared to Co_3_O_4_ samples, likely due to the smaller average size of NiFe_2_O_4_ (20 nm versus Co_3_O_4_’s 30–50 nm), allowing it to better fill pores within the CL and occupy more gaps among PTL fibers.

**Figure 3:**
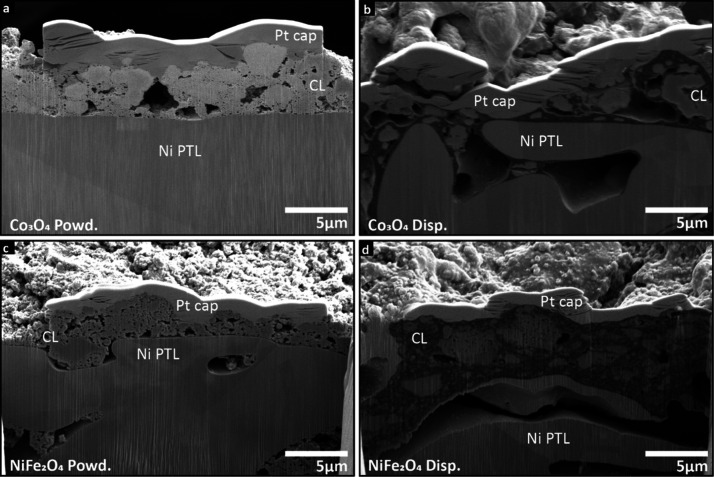
Cross-sectional SEM images of the pristine anodes with Versogen (a, c) powdered and (b, d) dispersed ionomers. (a) and (b) are with Co_3_O_4_. (c) and (d) are with NiFe_2_O_4_.

Lower HFR values for the powdered samples were identified and resulted in lower ohmic losses in Figure 2c,d. First, the CL fabricated with powdered ionomers had a higher amount of small pores (Figure 3 and Figure S10a), which likely improved electrolyte transport, potentially allowing for an improved CL/membrane interface and improved membrane hydration. Second, using the powdered ionomer form during fabrication created catalyst particles that were distributed more continuously, which made for better electronic conduction across the CL/PTL interface. For the dispersed samples, SEM-EDX results (Figure S9b,d) indicated that the CLs were more heterogeneous with more catalyst agglomerates (μm in scale) encapsulated by the ionomer (Figure 3b,d). In cases where the CL/PTL interface was mostly covered by the ionomer, higher ohmic losses may have been due to an increased tortuosity of electron pathways. With a different ionomer, Gen2 had less ohmic losses, mainly due to the thinner membrane (60 μm).

The CLR was evaluated to provide information on the electronic and hydroxide conductivity of the electrodes (Figure S8). In Figure 2c,d, CLR overpotentials accounted for small amounts and showed a consistent trend that powder-fabricated anodes had lower CLRs than the dispersed ones. Although the hydroxide conductivity of the commercial ionomer (Versogen) was reportedly higher than Gen2,^23,38^ the CLR did not follow the trend, which suggested that hydroxide transport primarily relied on the electrolyte in a concentrated KOH environment, consistent with past studies.^33,37,39^ Lower CLRs of the powdered samples was likely due to higher electronic conductivity (in-plane and through-plane) during operation and consistent with ex situ results from *R*_sheet_ and ICR in Table 2, where both Co_3_O_4_ and NiFe_2_O_4_ with powdered ionomers had 2 orders of magnitude lower *R*_sheet_ than the dispersed-based anodes. With the powdered ionomer, the ex situ ICR measurement was reduced by 1.08 and 3.50 Ω cm^2^ for Co_3_O_4_ and NiFe_2_O_4_, respectively. Higher CLR values of the dispersed samples were possibly due to catalyst/ionomer agglomerates, which may have filled the gaps between catalysts, isolating and worsening the electronic conduction of the anode CL. SEM-EDX results in Figure S9 further suggest that catalysts were distributed more continuously in the samples with powdered ionomers involved during fabrication, while inhomogeneous catalyst agglomerates tended to occur with dispersed ionomers.

**Table 2:** *R*_sheet_ and ICR Results of Powdered and Dispersed Versogen Ionomers with Different Catalysts

catalyst and ionomer forms	*R*_sheet_ (Ω/square)	ICR (Ω cm^2^)
Co_3_O_4_ Powd.	3.1 × 10^4^	0.45
Co_3_O_4_ Disp.	2.0 × 10^6^	1.53
NiFe_2_O_4_ Powd.	8.8 × 10^5^	0.82
NiFe_2_O_4_ Disp.	3.8 × 10^7^	4.32
IrO_2_ Powd.	1.5 × 10^4^	0.10
IrO_2_ Disp.	1.7 × 10^4^	0.44

By using the powdered ionomer with Co_3_O_4_, EIS at 1.5 V (Figure 4) showed that the *R*_ct_ decreased by 0.18 Ω (Versogen) and 0.11 Ω (Gen2), compared to the dispersed ionomer. The ECM results (summarized in Table S1) revealed that the anodes fabricated with the powdered ionomer had lower *R*_ct_ and higher capacitance values, suggesting an improved charge transfer process and that the electrochemical active area was higher. The corresponding DRT results at 1.5 V showed that the main relaxation process was located around the time constant (τ) in the range of 10^–2^ to 10^0^ s (16–0.16 Hz), which was attributed mostly to the OER charge transfer process.^31,40^ The sluggish features and multiple charge transfer steps of OER are well-known in water electrolysis.^41^ Additional peaks with τ < 10^–2^ may be related to the electrical double layer at the membrane/CL interface; these peaks may also be due to reaction kinetics since 1.5 V was in the predominantly kinetic region (see Figure S7).^11^ Although the HER and OER contributions may have overlapped within these peaks and were not distinguishable in DRT, the differences in EIS and DRT results were attributed to OER because the cathodes in each test were identical. To confirm the HER contribution, powdered and dispersed ionomers were used at the cathode with an identical anode material (Co_3_O_4_ Vers Powd.). The results (Figure S12) showed that the powdered ionomer had higher kinetic losses and larger *R*_ct_ in EIS at 1.5 V than the dispersed form when it was applied at the cathode. DRT (Figure S12d) also demonstrated two noticeable kinetic peaks with the powder-fabricated cathode, which implied that the dominant peak in Figure 4c likely resulted from the overlap of the OER and HER peaks. How the DRT peaks evolved with potential was evaluated (see Figure S4), where peaks with τ = 2 × 10^–4^ to 4 × 10^–1^ s decreasing with increasing voltage indicated the kinetic processes, while peaks with τ = 3 × 10^–5^ to 1 × 10^–4^ s could have been related to hydroxide and electronic conductivity throughout the CL.^24,31,40^ The kinetic-related peaks decreased when the anode was fabricated with a powdered ionomer, which likely improved site-access and charge transfer processes through better catalyst/ionomer distribution. The mass transport processes were less dominant at low potential, but may contribute either to the primary peak or to the right of the main kinetic peak with a slower τ (4 × 10^–1^ to 1 × 10^1^ s) in Figure 4c–f. From voltage breakdown analysis in Figure 2c, Co_3_O_4_ with a dispersed ionomer had higher overpotentials from other losses (mass transport), which may have shifted the main DRT peak to a higher range of time constants in Figure 4c,e because mass transport generally has a higher time constant than kinetic processes.

**Figure 4:**
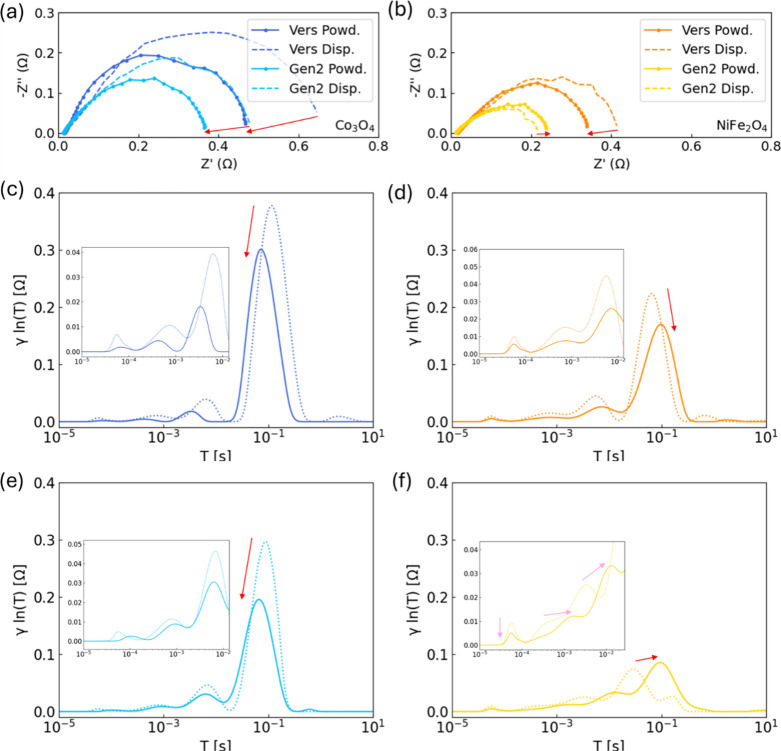
EIS at 1.5 V with (a) Co_3_O_4_ and (b) NiFe_2_O_4_. DRT results for Co_3_O_4_ and (c) Versogen and (e) Gen2 ionomers. DRT results for NiFe_2_O_4_ and (d) Versogen and (f) Gen2 ionomers.

Changes of the local pH during water splitting have been reported, which may result in catalyst reconstruction and dissolution due to an unstable oxide state.^39,42^ Cobalt oxyhydroxide has been identified as the active species with faster electron transfer and high stability, especially with a high-spin state of Co^3+^ or a higher oxidation state of Co.^41,43,44^ A more porous electrode structure created by the powdered ionomer might improve site-access and catalyst utilization, spread out operational stressors, and avoid possible OER hot spots that could decrease the pH in small portions of the electrode. The powder-fabricated CL may have had less local pH variation, and thus, the active intermediates during OER with Co_3_O_4_ might have been more stable than in the dispersed samples. Co_3_O_4_ fabricated with a powdered ionomer further had faster kinetic relaxation processes, which was likely attributed to better site-access to the amorphous layer (more active) around Co_3_O_4_ particles, facilitating faster and more efficient electron transfer of the OER. A higher degree of homogeneous utilization of Co_3_O_4_ could lessen structural stress and retain Co_3_O_4_ in the CL, which could be beneficial for crystalline structure reversibility of Co_3_O_4_.^45^

Similar EIS trends for NiFe_2_O_4_ with the Versogen ionomer can be identified in Figure 4b, where the *R*_ct_ reduced by 0.08 Ω with the powdered ionomer. Figure 4d further shows that the reaction kinetics also improved with less intensive peaks. The main peak (τ = 2 × 10^–2^ to 3 × 10^–1^ s) in the powdered sample was smaller and slower than in the dispersed-based anode for NiFe_2_O_4_. Better electronic conduction between catalyst agglomerates with the powdered ionomer was further suggested by the peak with the lowest τ (7 × 10^–5^ s). For the Gen2 ionomer, the *R*_ct_ slightly increased by 0.02 Ω in Figure 4b. Although the trend at 1.5 V was different from other samples, NiFe_2_O_4_ with the powdered Gen2 ionomer had a lower *R*_ct_ at higher voltages (*V* > 1.5 V in Figure S13). In Figure 4f, the main kinetic peaks (solid and dotted lines) were similar in height, and while the difference between Gen2 powdered and dispersed ionomers was not significant, the dispersed sample appeared to have more distributed peaks (two time constants at 2.6 × 10^–3^ and 1.8 × 10^–1^ s) than the powdered sample (one peak at 1 × 10^–1^ s). The EIS and DRT results at 1.7 V (Figure S13) demonstrated that the dispersed-based anode had a lower electronic conductivity, less homogeneous charge transfer rates, and slightly higher mass transport losses. In the CV results (Figure S14), although the shape can be influenced by the oxidation of PTLs and flow fields, the results suggested that the anode CL fabricated with the powdered ionomer might have a higher electrochemical surface area from the more pronounced redox features. With the powdered ionomer during electrode fabrication, a higher number of active sites (evidenced by higher *C* values from ECM in Table S1) allowed for more efficient charge transfer processes. The performance improvement of NiFe_2_O_4_ was less pronounced than that of Co_3_O_4_, which was possibly due to the higher intrinsic surface area of NiFe_2_O_4_.^9^

#### 3.2.2 PGM Catalyst

The current responses of IrO_2_ in Figure 5a were less promising compared to those of non-PGM catalysts; however, the powdered ionomer fabricated in the anode CL effectively reduced kinetic and CLR losses (Figure 5b), aligning with the results observed for non-PGM catalysts. With a Versogen membrane and ionomer, IrO_2_ had lower kinetic losses than Co_3_O_4_ in Figure 2c, likely due to its higher catalyst activity and electronic conductivity. However, compared with non-PGM catalysts, IrO_2_ had higher overpotentials from other losses. This may have been attributed to its smaller particle/agglomerate size, which could have changed the distribution of the catalyst, ionomer, and pores in the CL, and thus, electron and ion transfer through the CL might have been affected. Moreover, IrO_2_ as a catalyst may potentially cause ionomer degradation during the break-in procedure due to a higher reactivity and the increased number of sites, resulting in CL structure changes and partial delamination that may have affected mass transport.^9,10,46^

**Figure 5:**
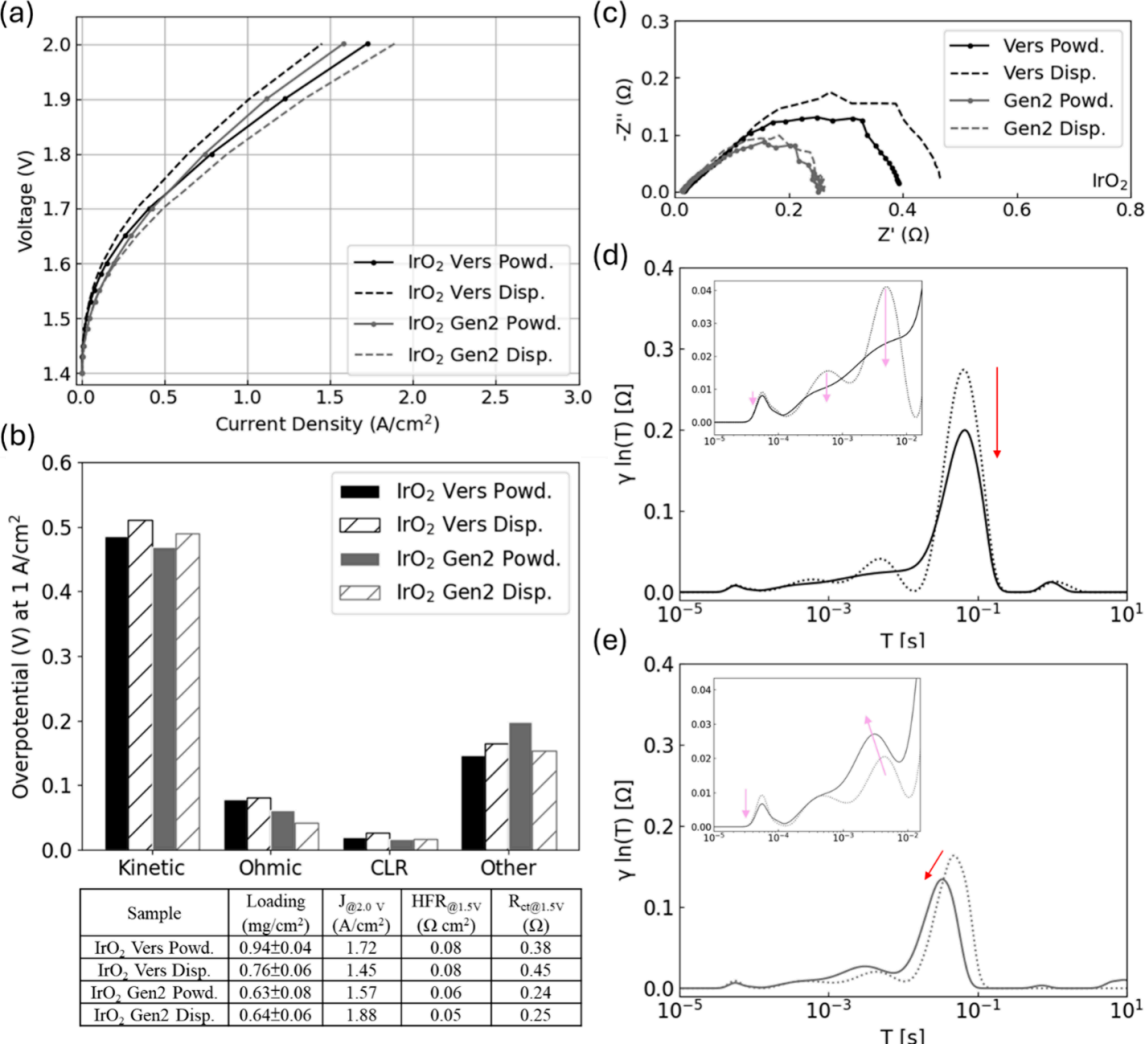
Polarization curves of powdered (Powd.) and dispersed (Disp.) ionomers from Versogen (Vers) and Gen2 with (a) IrO_2_ in anode catalyst layers. (b) Overpotentials from kinetic, ohmic, CLR, and other losses at 1 A/cm^2^ with IrO_2_. (c) EIS at 1.5 V. DRT results from (a): with (d) Versogen and (e) Gen2 ionomers.

For powdered and dispersed ionomers, the kinetic overpotential in Figure 5b showed an improvement of 27 mV with the Versogen powdered ionomer. The capacitance values obtained from ECM (Table S1) were 0.37 and 0.36 F for the powdered and dispersed samples, respectively. The decrease in *R*_ct_ (Figure 5c and Table S1) observed with the Versogen powdered ionomer may have been related to improved catalyst/ionomer distribution and reduced ionomer–contaminant effects due to fewer hot spots, resulting in a compressed OER charge transfer peak in Figure 5d. For Versogen ionomers, as seen with non-PGM catalysts, powdered ionomers improved performance by enhancing interfacial contact (less ohmic losses), providing more continuous electronic conduction (lower CLR), and enabling favorable transport pathways. SEM-EDX results in Figure S9 showed that the anode fabricated with the Versogen powdered ionomer had improved interfacial contact with more continuous Ir signals at the CL/PTL interface and potentially higher catalyst utilization due to an improved Ir particle distribution and a more homogeneous CL. The dispersed sample showed that Ir was mostly covered and isolated by ionomers. Table 1 further shows that IrO_2_ with the Versogen dispersed ionomer had approximately 2.4 × 10^3^ Ω/square and 0.34 Ω cm^2^ higher R_sheet_ and ICR, respectively, than the powdered sample.

The powdered Gen2 ionomer also had lower kinetic losses (22 mV difference) than the dispersed. Although *R*_ct_ in EIS at 1.5 V was similar (approximately 0.24 Ω), the Gen2 dispersed ionomer exhibited a higher double-layer capacitance (0.38 F from ECM in Table S1) than the powdered one (0.34 F). This difference may have been attributed to increased Ir aggregation with the Gen2 powdered ionomer, potentially causing higher transport losses, as shown in Figure 5b. Additionally, the DRT results indicated slower polarization processes (peaks distributed within 4 × 10^–1^ to 2 × 10^1^ s), leading to a greater impedance when IrO_2_ was combined with the Gen2 powdered ionomer.

### 3.3 Powdered Ionomer Content

Catalyst inks with varying ionomer contents influence the catalyst–ionomer distribution and ink homogeneity, leading to variations during the ink deposition process. These variations, in turn, influence the electrode morphology, including porosity, pore size, agglomerate distribution, and stability. Although the ink composition and the dispersion history of powdered and dispersed electrodes were different, the reproducible results in performance and morphology were verified through the comparison of CCS versus CCM, different catalysts, ionomers, and ionomer contents. Optimizing the ionomer content across various cell components can enhance performance and provide insights into catalyst–ionomer interactions. A high ionomer content can insulate the catalyst particles, reduce catalyst utilization, decrease electronic conductivity, and disrupt mass transport pathways. Conversely, a low ionomer content can lead to reduced lower ionic conductivity and increase CL delamination due to insufficient adhesion.^12,15,47^ Figure 6a shows the polarization curves of powder-fabricated electrodes with ionomer contents ranging from 1 to 30 wt % at the beginning of life (BOL) after the conditioning steps. Figure 6d–h includes EIS at BOL and end of life (EOL) after a 2V hold for 12.5 h.

**Figure 6:**
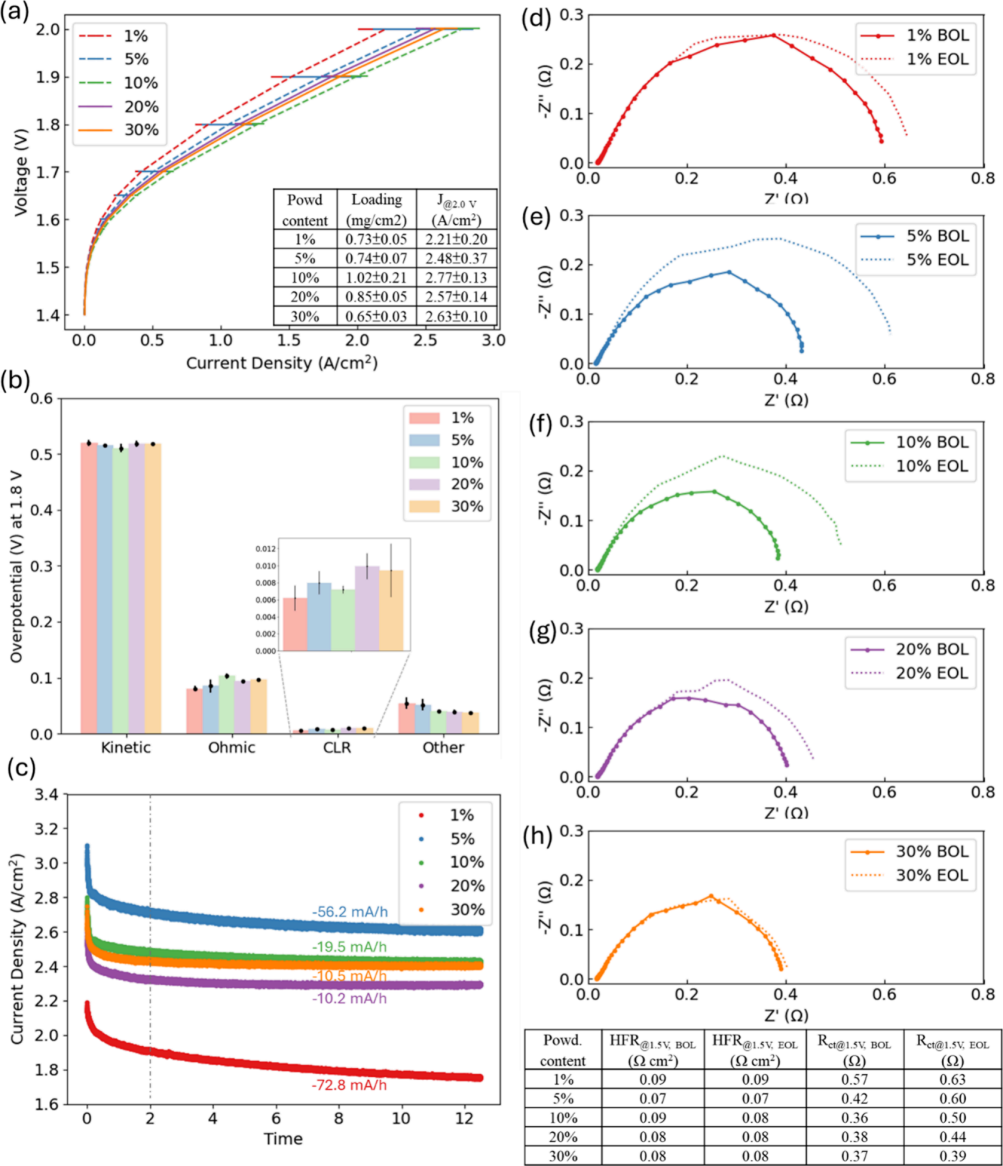
(a) Polarization curves of 1–30 wt % Versogen powdered ionomers with Co_3_O_4_ in anode catalyst layers. (b) Overpotentials from kinetic, ohmic, CLR, and other losses at 1.8 V from (a). (c) Current responses at 2 V held for 12.5 h. (d–h) EIS at 1.5 V with 1–30 wt % powdered ionomers at BOL and EOL.

#### 3.3.1 Optimum Performance

For the BOL performance, similar to a previous study on a Versogen dispersed ionomer,^15^ a 10 wt % ionomer had the best performance (2.76 A/cm^2^ at 2 V). Figure S15 shows the relationship of *V*_HFR‑free_ and current, demonstrating better kinetic performance as the ionomer content increased from 1 to 10 wt %. Specifically, the kinetic overpotential decreased by 18 mV at 1.8 V (Figure 6b), potentially due to better dispersion capability with a higher powdered ionomer content in the ink. EIS at 1.5 V in Figure 6f also showed that 10 wt % had a lower *R*_ct_ (0.37 Ω) at the BOL. At ionomer contents higher than 20 wt %, kinetic losses in Figure 6b and the *R*_ct_ in Figure 6g,h slightly increased, possibly because excess ionomer in the anode CL blocked active reaction sites. Ohmic overpotentials in Figure 6b showed the increasing trend with increasing ionomer contents (1–10 wt %), which indicated that the electrical network in the anode CL might have been less continuous (less electrical conduction) with a higher ionomer content. Nonuniform ionomer distribution at the CL/PTL or CL/membrane interface may result in higher ohmic losses when the ionomer amount increases from low to middle levels. However, at higher ionomer contents (20 and 30 wt %), CL exhibited lower ohmic losses compared to 5 and 10 wt %. This improvement was likely due to more efficient KOH transport, enhanced hydration of the membrane, increased membrane hydration, and better membrane/CL contact resistance. Assuming that the hydroxide conductivity is less dependent on the ionomer content in a concentrated KOH environment,^33^ lower electrical conductivity with higher ionomer contents is corroborated by CLR overpotentials (as approximated in Figure S15). For other losses, 1 and 5 wt % had higher overpotentials (around 75 mV) at 1.8 V, while the CL with a 10–30 wt % ionomer was less than 60 mV. With little ionomer content in the electrode, the morphology may have been more compacted and thus resulted in higher mass transport losses.^15^ With an additional powdered ionomer added in the anode (up to 30 wt %), the ink stability was likely improved, promoting the formation of a more porous and well-connected electrode structure on the PTL, which in turn can facilitate better mass transport paths for KOH and oxygen.

#### 3.3.2 Degradation Mechanisms

The ionomer content and its distribution in the anode CL are critical for not only cell performance but also electrode stability. During longer operation (12.5 h), the degradation rate was calculated from 2 to 12.5 h to exclude the initial current drop primarily due to short-term passivation, as shown in Figure 6c. These tests revealed higher degradation rates (72.8 and 56.2 mA/h) at low ionomer contents (1 and 5 wt %), potentially caused by a weakened CL structure and delamination and heterogeneous potential distribution (hot spots) that may have accelerated ionomer oxidation and catalyst dissolution. Moreover, a reduced number of electrochemical active sites and inhomogeneous catalyst utilization due to the loss of catalysts at low ionomer contents may have led to increased degradation. From 5 to 30 wt %, EIS at 1.5 V in Figure 6e–h shows a clear trend that higher ionomer contents helped mitigate degradation in kinetic properties at the EOL (less changes in *R*_ct_). The ionomer content with 1 wt % in Figure 6d had a smaller increase in *R*_ct_ at the EOL possibly because the degradation might have happened during the conditioning step (2 V for 2 h). During electrochemical diagnostics, Co_3_O_4_ underwent redox transitions, with parts of the catalyst transition being between crystalline and amorphous states. After 12.5 h at 2 V, the CL likely had fewer active sites, as Co_3_O_4_ became more crystalline and less active. Inhomogeneous local current and bubble coverage within the CL caused increased mechanical stress, deteriorating cell performance, and inducing failure, such as short circuits (Figure S17). In the repeated runs, the anode CL with an ionomer content <10 wt % tended to fail due to shorting, while those with 20–30 wt % continued to perform through the complete testing protocol.

In a longer durability test (500 h) in Figure S18, Co_3_O_4_ with a 20 wt % powdered ionomer demonstrated stable performance with a 29.2 μA/h decay rate at 2 V. Extended operation further resulted in increases in the HFR and CLR, accompanied by an improvement in kinetics. While operation likely results in PTL (and CL) passivation and increases in the in- and through-plane electronic resistances (HFR, CLR), membrane changes may affect the HFR as well. The kinetic improvement, however, indicated that ionomer oxidation and catalyst delamination were likely not the primary driver of performance losses, at least at a moderate ionomer content (20 wt %). The powdered ionomer form at low contents, however, may lessen the catalyst–ionomer interface to point contacts, which may negatively impact stability, consistent with shorting at ionomer contents <10 wt %. Although applying a powdered ionomer in the anode CL can increase the catalyst activity by creating higher amounts of active area, the uniform and robust CL must be investigated with an appropriate amount of catalyst loading and ionomer content for longer-term operation.

## 4 Conclusions

The use of powdered and dispersed ionomers created differences in electrode properties and AEMWE performance and durability through the use of different ionomer forms and ink properties. While inks and electrodes were prepared by controlled processes, the ink chemistries were not identical, and solvent-additive differences and dispersion processing history may contribute to differences in ink rheology and the resulting electrodes. This study, however, robustly demonstrates a practical morphological difference between electrodes and an AEMWE performance and durability improvement based on the as-supplied ionomer form.

Fabricating the anode with powdered ionomers resulted in a significant performance improvement in AEMWE with 1 M KOH compared to using the dispersed form, with 46.3% (Versogen) and 20.0% (Gen2) higher current responses at 2 V for a Co_3_O_4_ catalyst. The results from in situ and ex situ characterization methods demonstrated that the powdered ionomer plays a role during fabrication in providing uniform morphologies in terms of the distribution of the catalyst, ionomer, and pores in the anode CL and resulted in improved electronic conductivity, reaction kinetics, and mass transport. SEM-EDX results revealed that the powdered ionomer contributed to forming a more porous CL structure, enhancing the reaction active area, and improving mass transport properties. In contrast, electrode structures with dispersed ionomers appeared to exhibit reduced electronic conduction, potentially caused by the isolation of catalyst particles by nonconductive dispersed ionomer clusters. EIS analysis indicated that the powder-fabricated anodes effectively reduced kinetic, ohmic, and CLR losses for both PGM and non-PGM catalysts. For Co_3_O_4_ and NiFe_2_O_4_, the commercial ionomer (Versogen) in its powdered form consistently contributed to lower charge transfer resistances and higher capacitance values, suggesting more efficient kinetics and a higher electrochemical active area in the electrode. Optimizing the powdered ionomer content to 10 wt % might have improved the distribution of catalyst particles, enabling a continuous electronic network and more homogeneous catalyst utilization.

By using a powdered form of ionomers, an improvement of cell efficiency has been found along with reasonable stability. Studies here focused on operation in 1 M KOH due to the higher performances and lower durability losses compared to lower electrolyte concentrations or water operation. Operation in lower electrolyte concentrations, however, requires greater ionic transport from the ionomer, changing the catalyst layer property and ionomer distribution requirements. Lower electrolyte concentration therefore likely impacts the findings and the optimal powdered ionomer contents presented in this study.

Current AEMWE limitations include not only low efficiency but also an insufficient understanding of degradation processes. Catalyst–ionomer interactions play crucial roles in performance during extended operations. Hydroxide availability relies more on the ionomer in the electrode in a lower concentrated electrolyte; however, the voltage decay rate may increase significantly. Optimizing the ionomer content and its distribution with catalysts in the CL may mitigate ionomer oxidation and catalyst dissolution and improve the catalyst–ionomer interaction, thereby minimizing degradation processes during extended operation in a hydroxide-rich environment. This research underscores the advantages of using a powdered ionomer in the anode CL to strengthen cell performance and stability, which is critical to position AEMWE as a competitive technology.

## Supplementary Material



## Notes

Kramers–Kronig test; equivalent circuit model (ECM); EIS and DRT validation, nonfaradaic impedance (NFI) for catalyst layer resistance (CLR) estimation, and Tafel analysis; cross-sectional and top-down anodes from SEM-EDS; cathode contribution in EIS/DRT; CV; ECM, Tafel slope, and CLR results (PDF)
