# Supplementary material for: Advancing the Performance of Anion Exchange Membrane Electrolysis by Employing a Powder-Based Ionomer during Anode Catalyst Layer Fabrication
Source: ACS Appl Energy Mater. 2026 Feb 19;9(5):2611–23. doi: 10.1021/acsaem.5c03719 (PMC12976989; doi:10.1021/acsaem.5c03719)
Supplement: Supplementary file 1 [file ae5c03719_si_001.pdf]

Supporting Information

Advancing the Performance of Anion Exchange Membrane Electrolysis.by Employing Powdered-Based Ionomer during Anode Catalyst Layer Fabrication

Ai-Lin Chan[a], Arielle L. Clauser[b], Makenzie R. Parimuha[a], James L. Young[a], Joshua D. Sugar[b], Shaun M. Alia[a]*

[a] Chemical and Material Sciences Center, National Renewable Energy Laboratory, 15013 Denver West Parkway, Golden, 80401, Colorado, United States
[b] Sandia National Laboratories, Livermore, 94550, California, United States
*Email : Shaun.Alia@nrel.gov

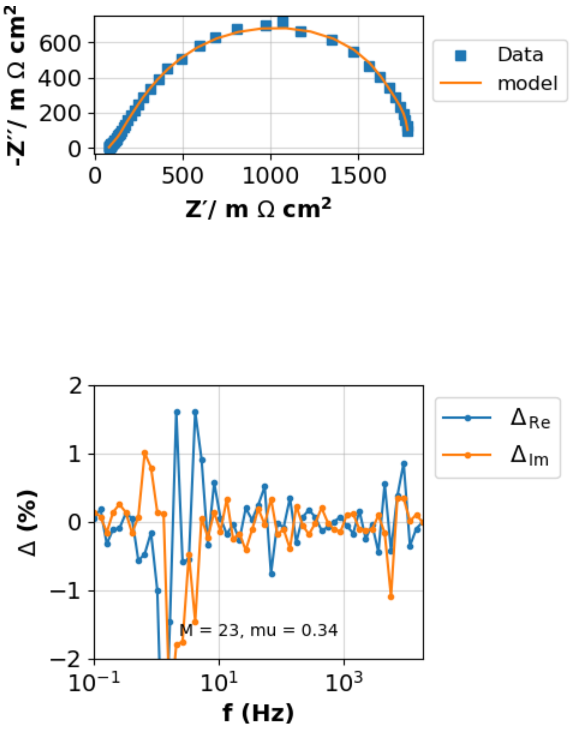

Figure S1. An example of KKT from EIS at 1.5 V. The Nyquist plot (top) and the residual values (Δ(%)) as a function of frequency (bottom). The amount of RC elements (M) in KK relation is 23. A factor (mu) was used to avoid over- and under-fitting during the KKT.

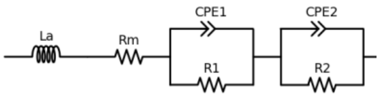

Figure S2. ECM configuration. R_m is ohmic resistance, $R_{ct,1}$ and $R_{ct,2}$ is the charge transfer resistance of slow and fast reaction, respectively; CPE_1 and CPE_2 are the constant phase element of the corresponding time constant, the impedance is in eqn(1). The capacitance can be estimated from CPE by eqn (2) and gives information on electrochemical active area between different samples.

$$Z_{CPE} = \frac{1}{Q(j2\pi f)^n} \tag{1}$$

$$C = Q(\omega_{max})^{n-1} \tag{2}$$

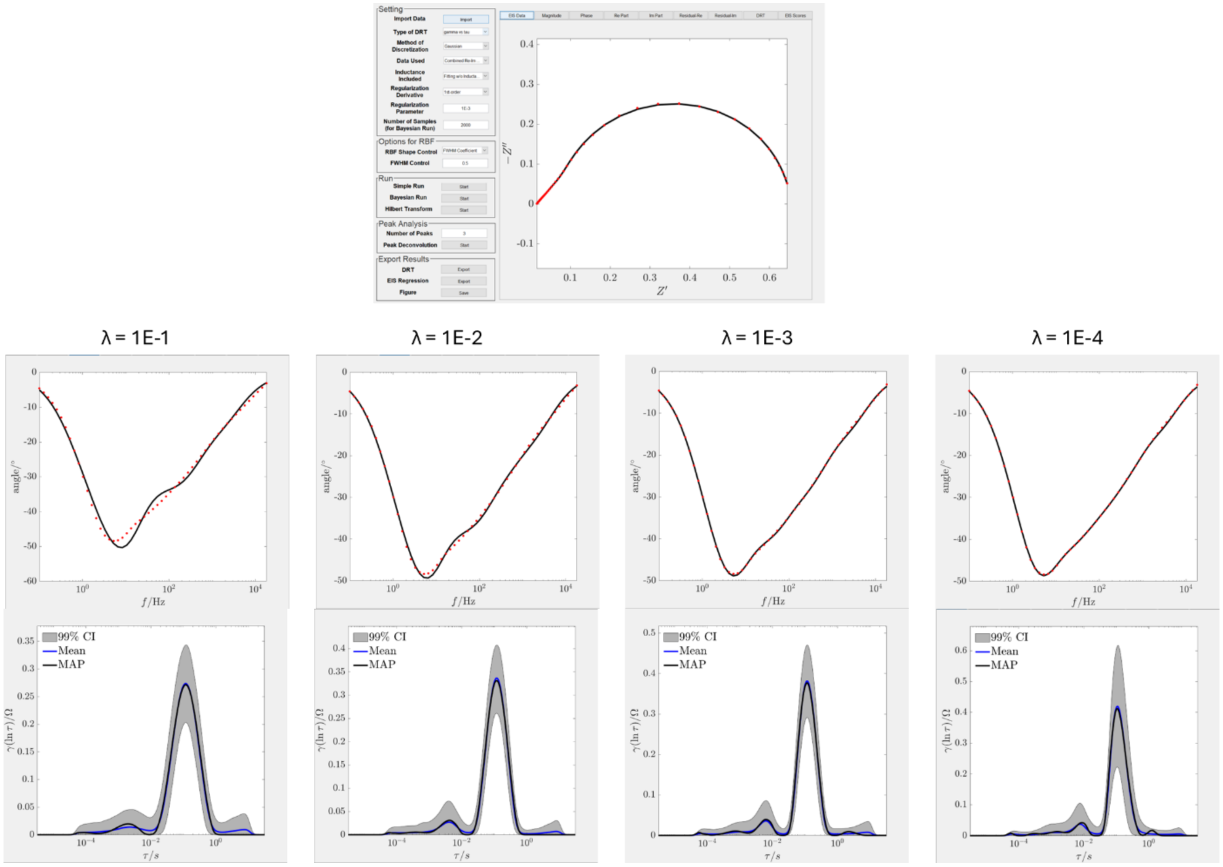

Figure S3. The input setting and parameters during DRT analysis in DRTools. The results of regularization parameter (λ) varied from 1E-1 to 1E-4. Experiment and modeled EIS data is in red points and black curve, respectively.

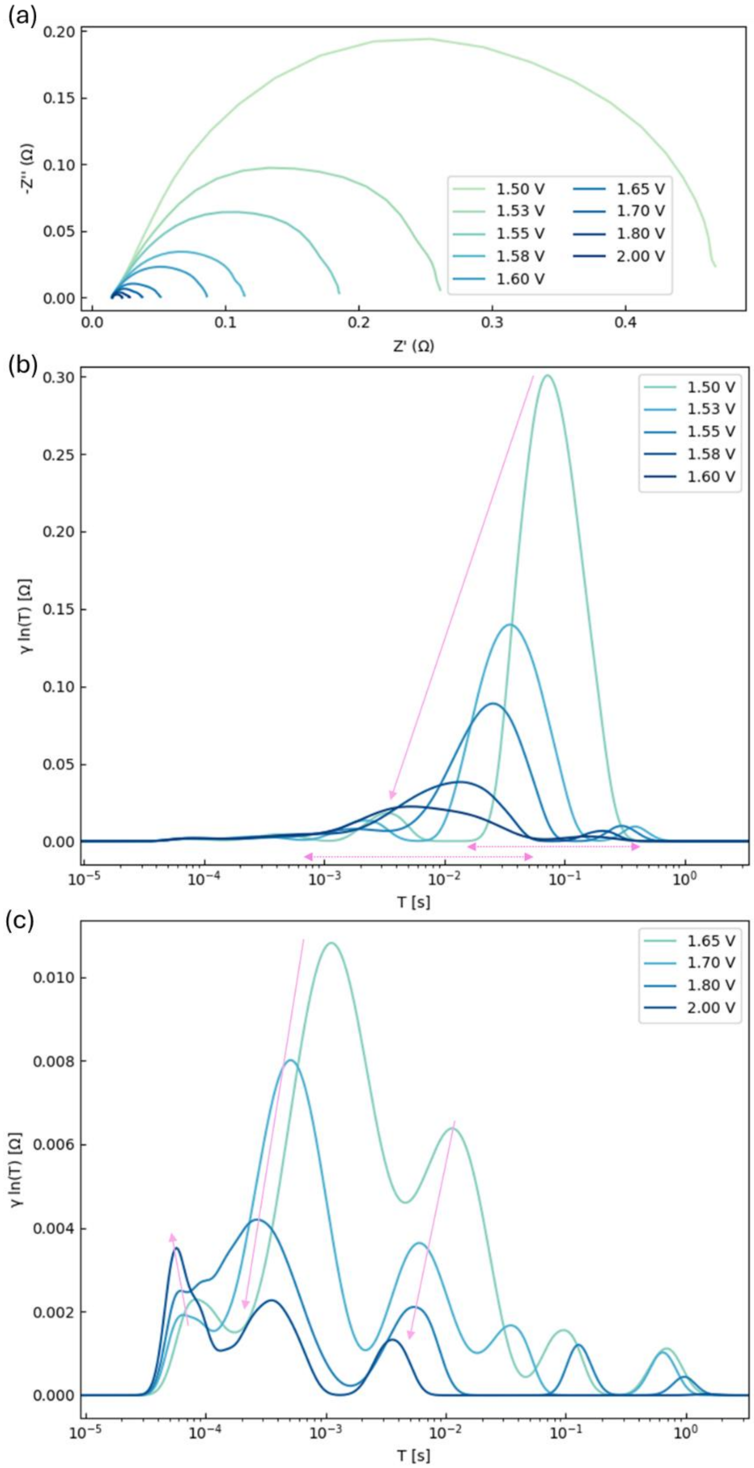

Figure S4. EIS and DRT results from 1.5 – 2.0 V with Co_3O_4 anode catalyst and powdered ionomer.

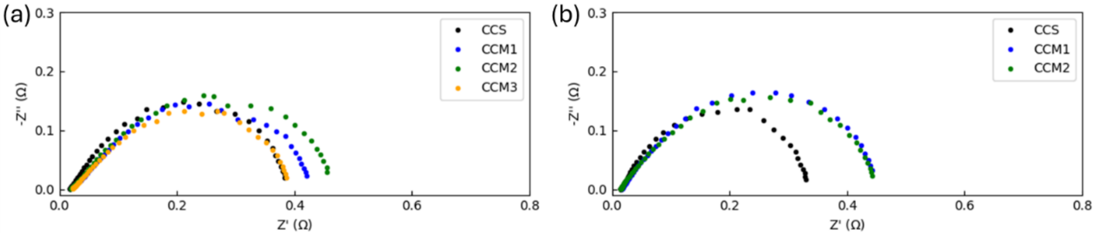

Figure S5. EIS at 1.5 V with different fabrication methods. (a) with dispersed ionomer. (b) with powdered ionomer.

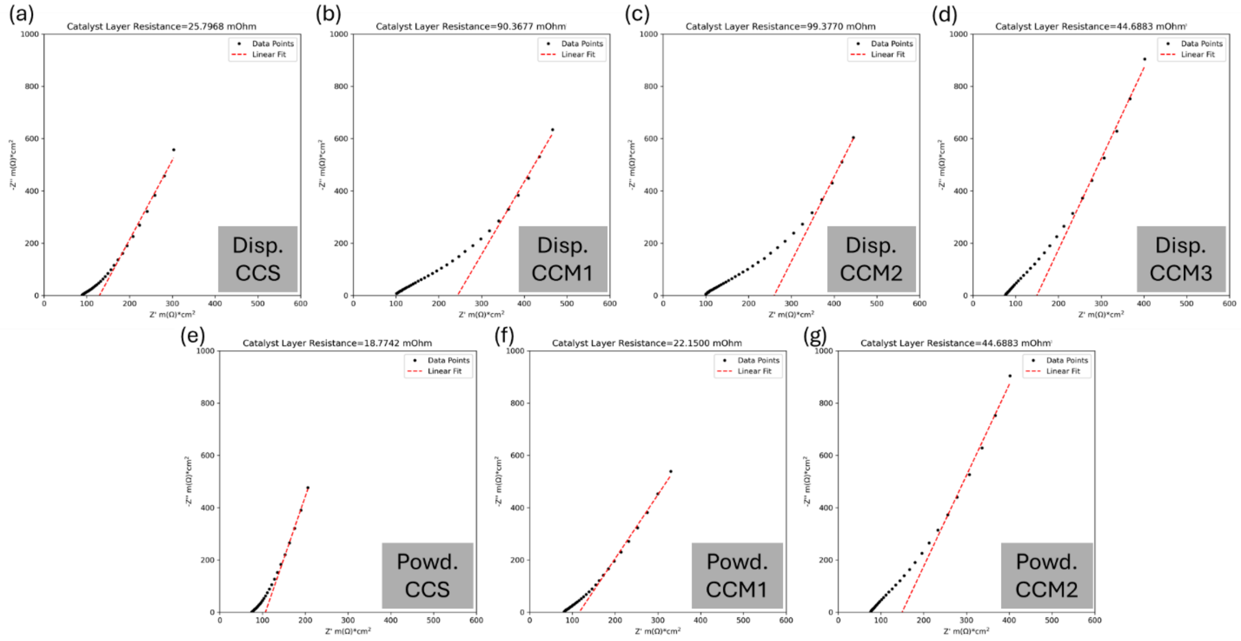

Figure S6. CLR approximation from NFI measurement with different fabrication methods. (a-d) with dispersed ionomer. (e-g) with powdered ionomer.

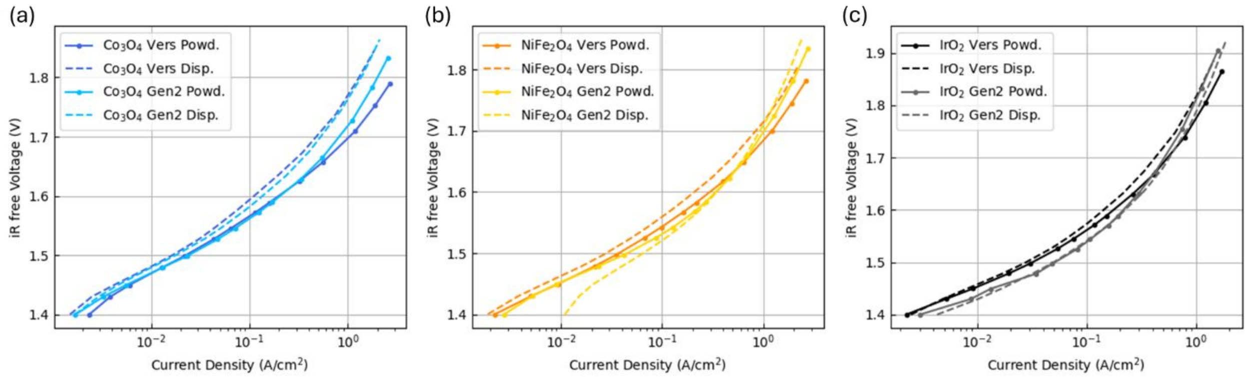

Figure S7. The relation of $V_{iR\text{-}free}$ and current density with the anode catalyst (a) Co_3O_4, (b) $NiFe_2O_4$ and (c) IrO_2.

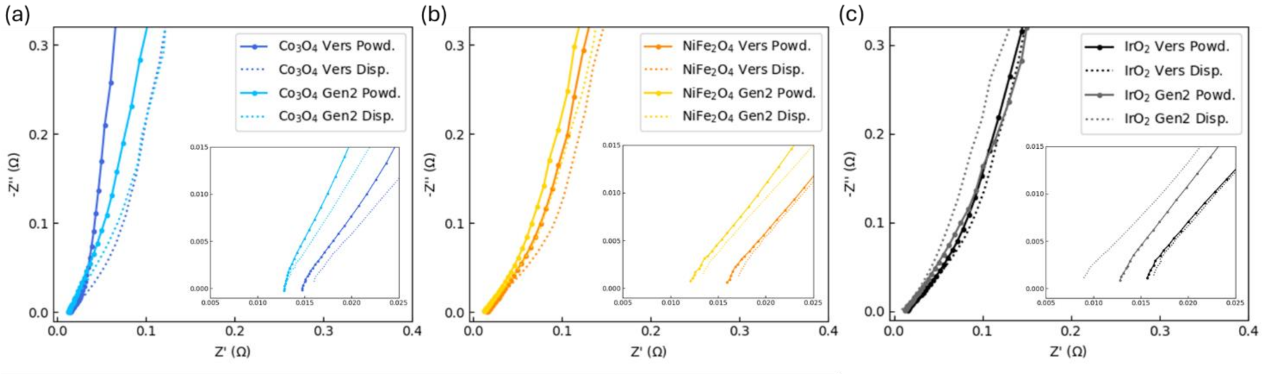

Figure S8. NFI with the anode catalyst (a) Co_3O_4, (b) $NiFe_2O_4$ and (c) IrO_2.

To study the ionomer phase in the anode by SEM-EDX, samples with Versogen ionomer were stored in 5 M KI (Sigma Aldrich, 99%) for 72 h for ion exchange. The samples were placed in foil to avoid KI photodegradation. After 72 h, the samples were rinsed with DI water for three times. The electrodes were then saved in water for 24 hours to remove any remaining K salts, and air dried for 24 hours before SEM sample preparation.

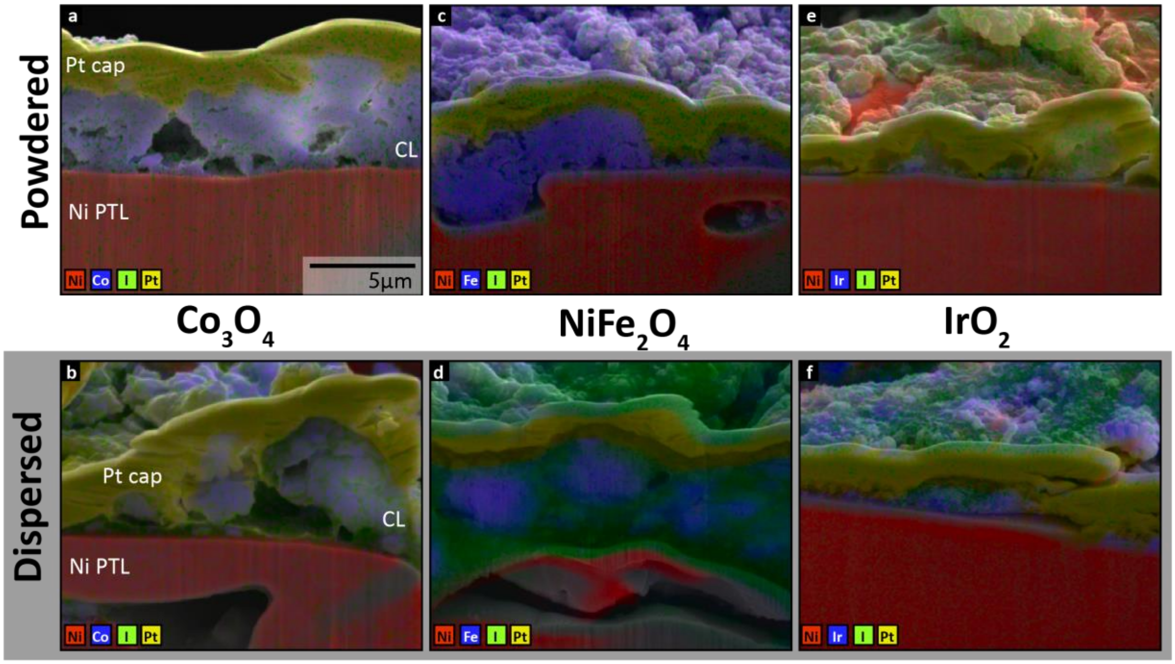

Figure S9. SEM images of FIB cross sections with overlaid EDX maps of pristine samples with Versogen powdered and dispersed ionomer with (a-b) Co_3O_4, (c-d) $NiFe_2O_4$ and (e-f) IrO_2 metal oxide catalysts. The metal oxide in each image is colored blue for comparison, the ionomer was exchanged for Iodine, differences in agglomeration of catalyst and ionomer can be seen between the powdered and dispersed samples. Protective Pt caps used during FIB cross sectioning are colored yellow.

(1)

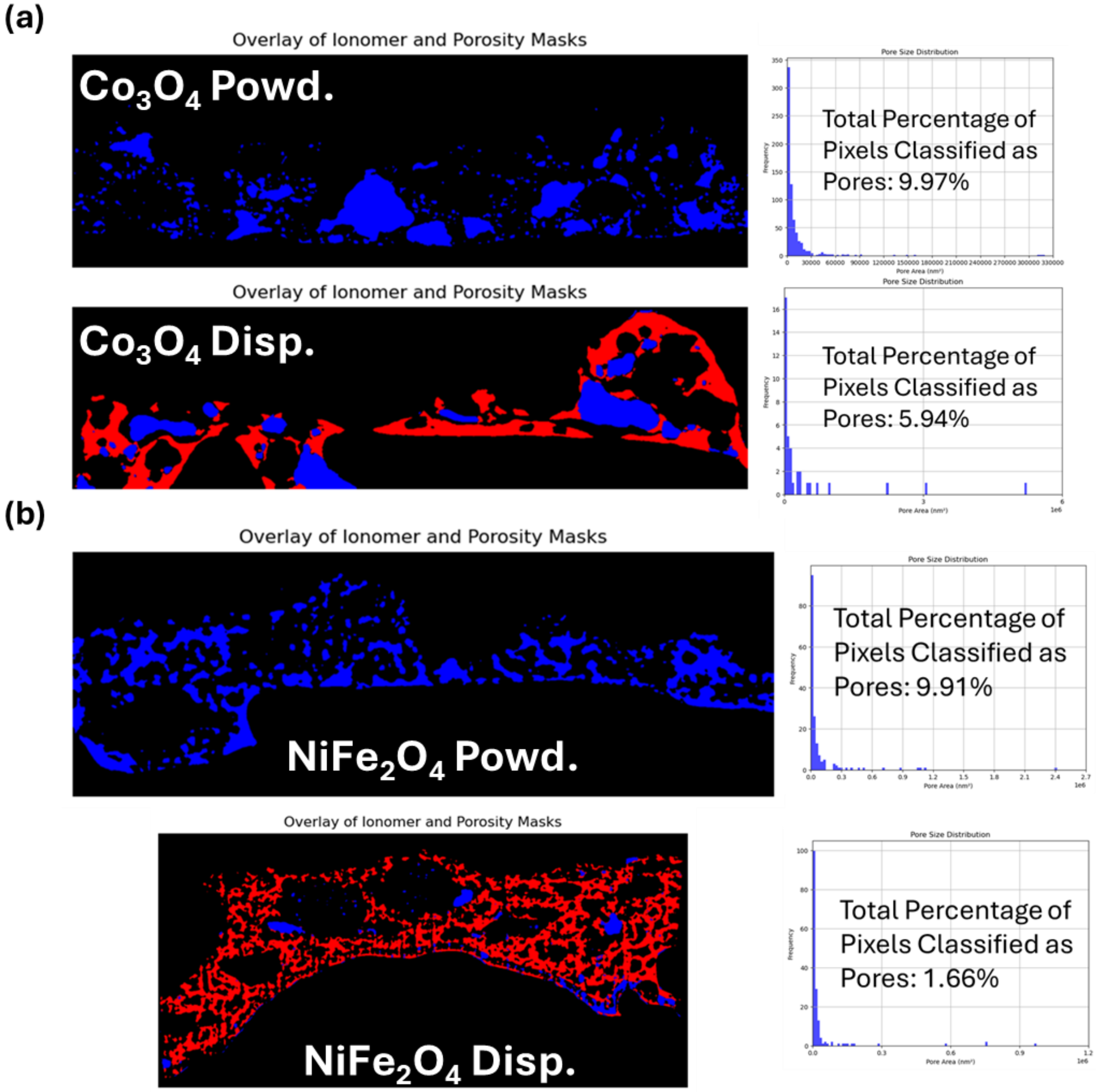

(2) CL Porosity Calculation (Co_3O_4 as an example):

$$\text{CL density: } (1 * \text{catalyst} + 0.2 * \text{ionomer}) / (1 + 0.2) = (6.11 + 0.2 * 1) / 1.2 = 5.26 \frac{g}{cm^3}$$

$$\text{CL loading: } (0.7 * 240.8 / (58.93 * 3)) * 1.2 = 1.14 \frac{mg}{cm^2}$$

$$\text{Theoretical thickness} = \text{CL loading} / \text{CL density} = 2.18\ \mu m$$

$$\text{Porosity} = \text{Theoretical thickness} / \text{Actual thickness} = 8.79\ \%$$

Figure S10. Porosity was estimated by two means: (1) SEM images processing from Figure S9. Ionomer (red) and pores (blue) in the anode catalyst layer with (a) Co_3O_4 and (b) $NiFe_2O_4$. (2) via mathematical calculation with CL density, catalyst loading and thickness.

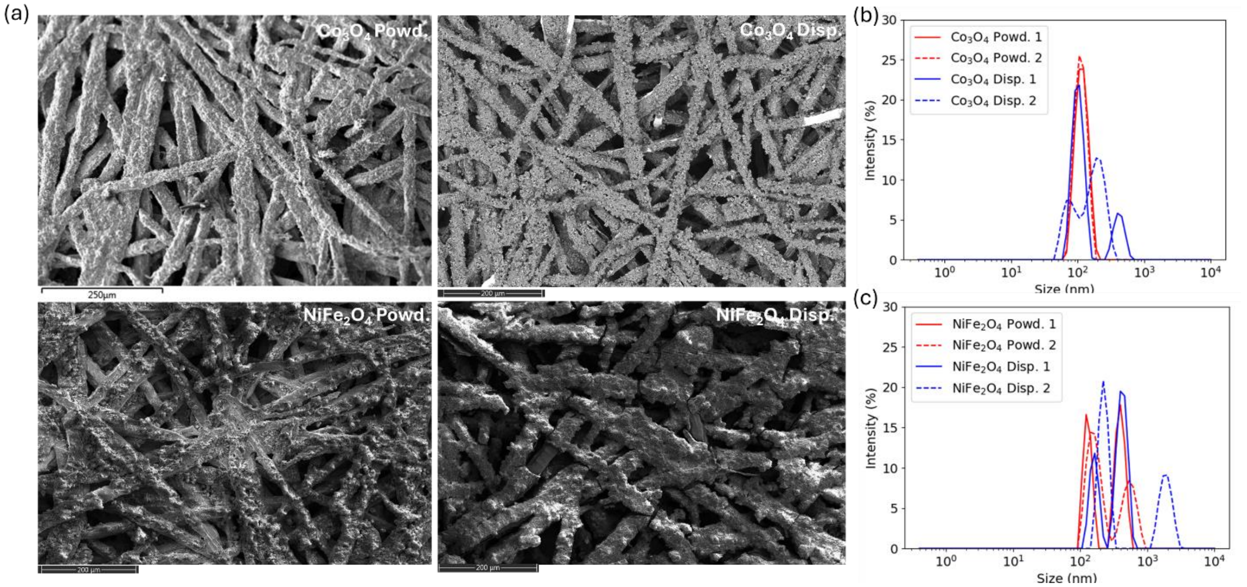

Figure S11. (a) Top-down images of Versogen powdered and dispersed ionomer with Co_3O_4 and $NiFe_2O_4$ and (b – c) the DLS results.

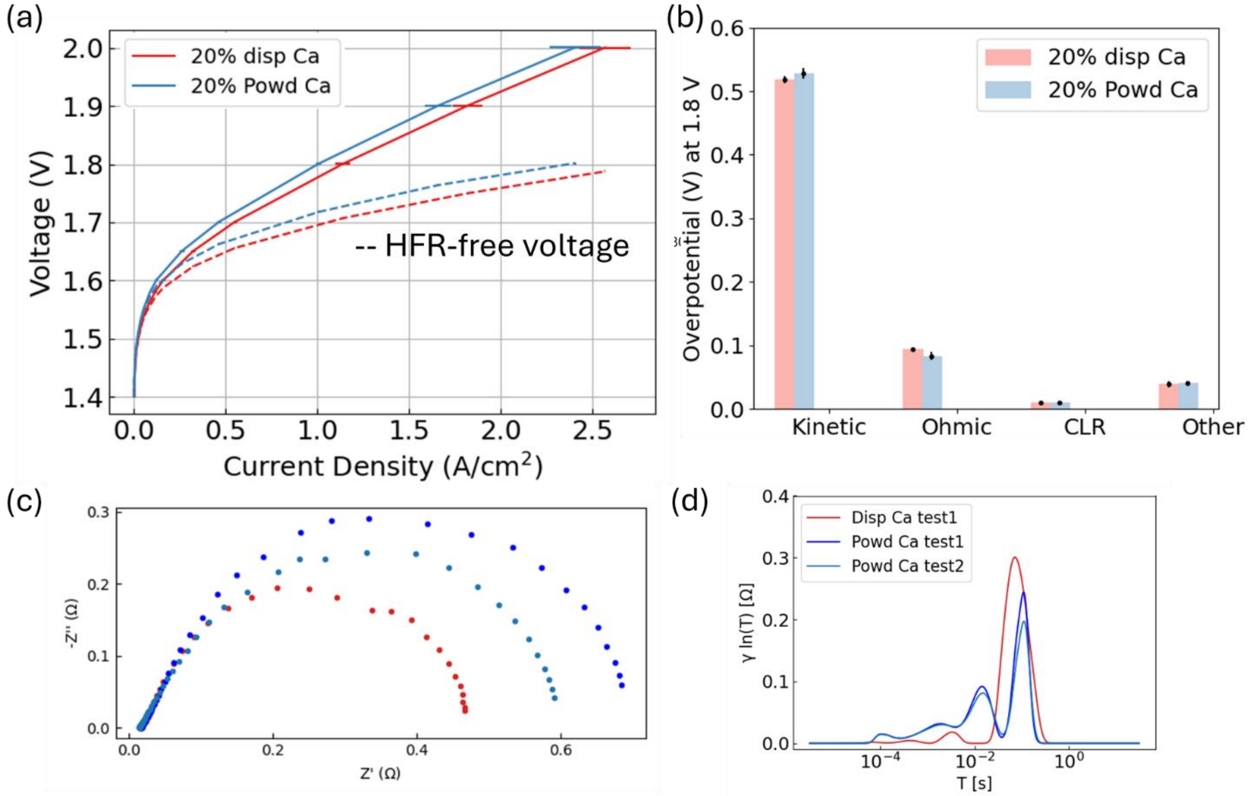

Figure S12. (a) Polarization curves and HFR-free voltages (--) of powdered and dispersed Versogen ionomers (20 wt%) with Pt/C at the cathode (Ca) and Co_3O_4 with 20 wt% powdered Versogen ionomers at

the anode. (b) Overpotentials from kinetic, ohmic, CLR and other losses at 1.8 V from (a). (c) EIS at 1.5 V. DRT results from (c).

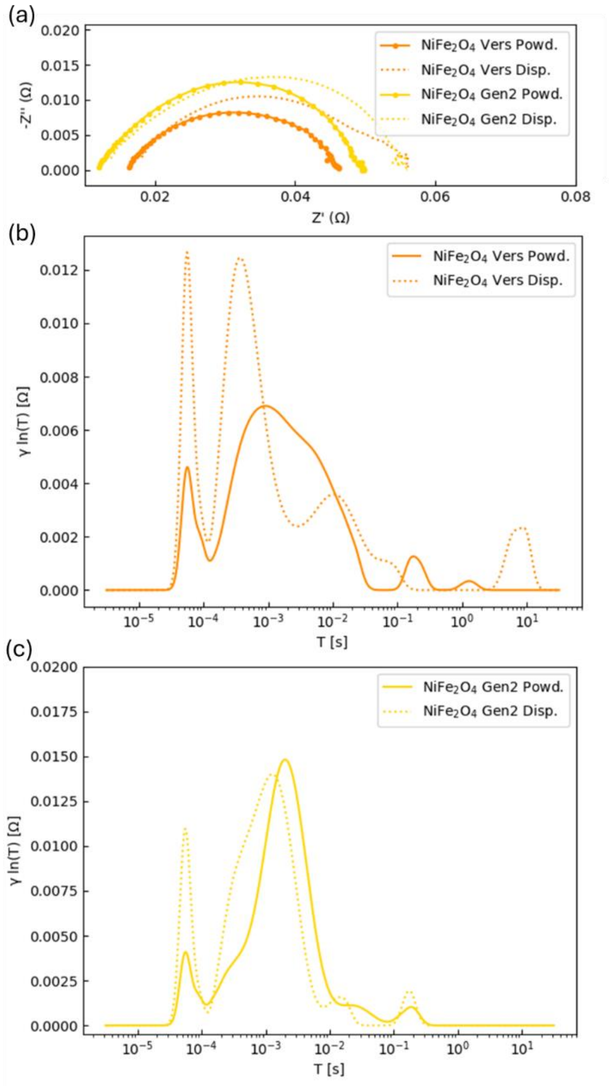

Figure S13. $NiFe_2O_4$ with powdered and dispersed ionomers. (a) EIS at 1.7 V. DRT results at 1.7 V of (b) Versogen and (c) Gen2 ionomers.

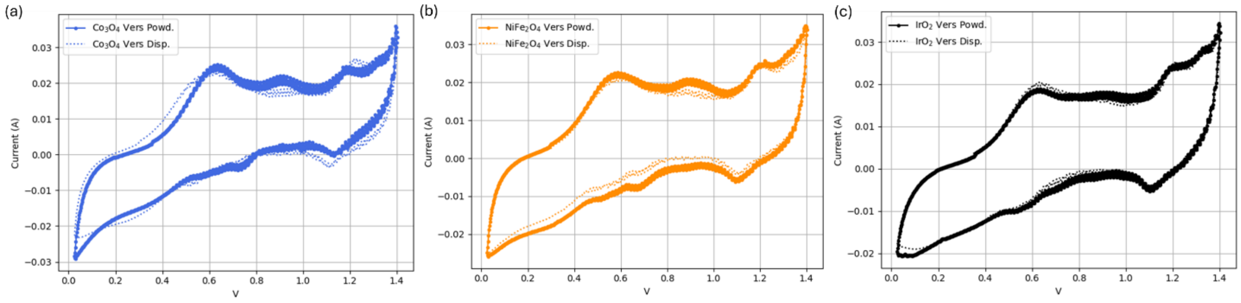

Figure S14. CV (20 mV/s) with the anode catalyst (a) Co_3O_4, (b) $NiFe_2O_4$ and (c) IrO_2.

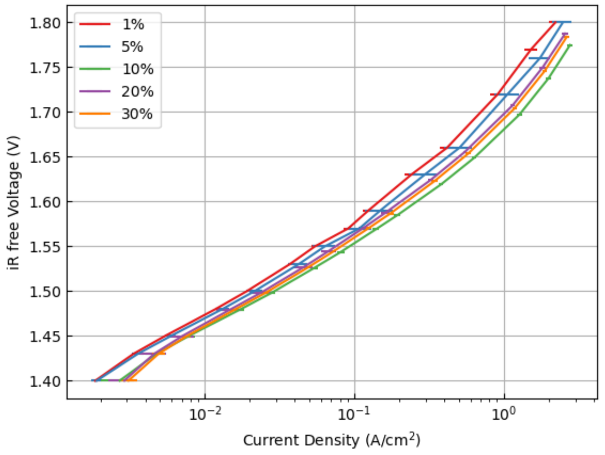

Figure S15. The relation of $V_{iR\text{-}free}$ and current density with Co_3O_4 anode catalyst with 1 – 30 wt% of powdered ionomer contents.

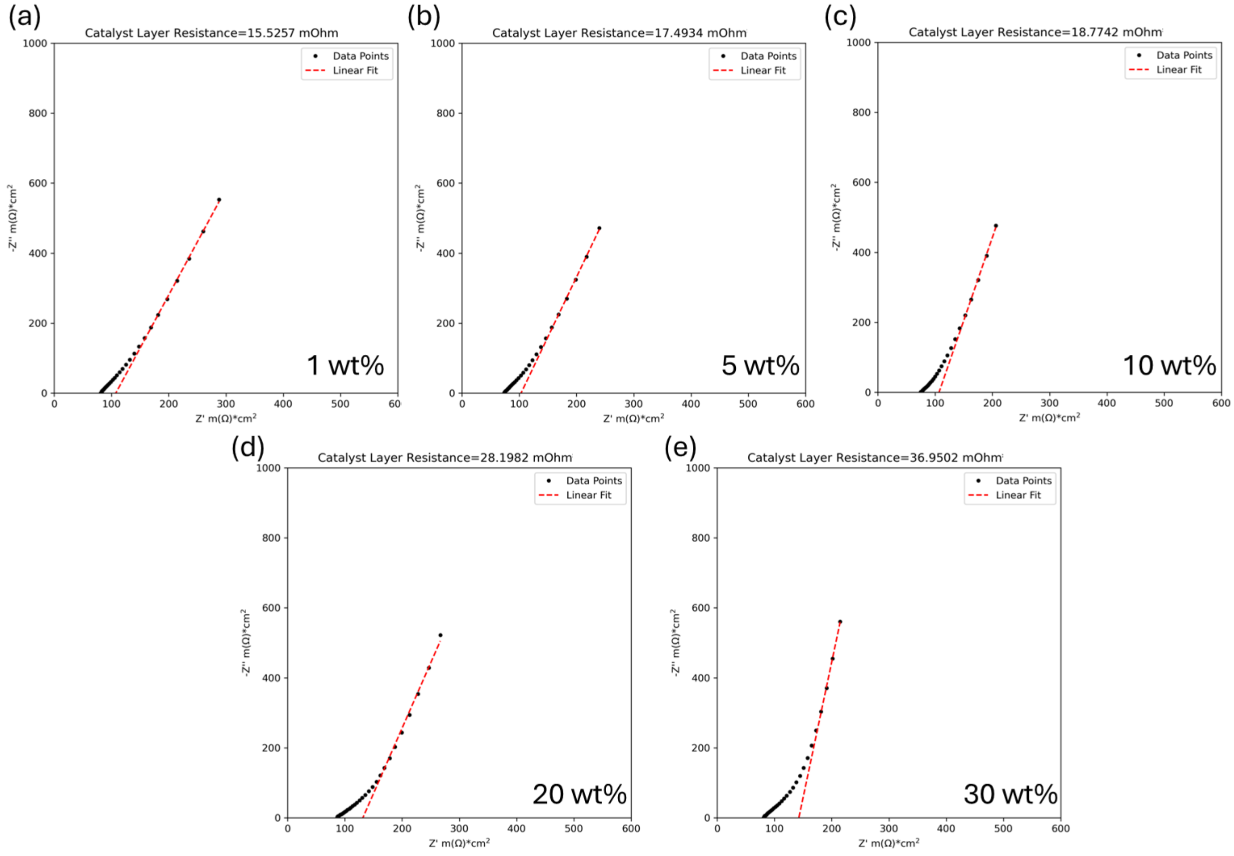

Figure S16. NFI with Co_3O_4 anode catalyst with 1 – 30 wt% of powdered ionomer contents.

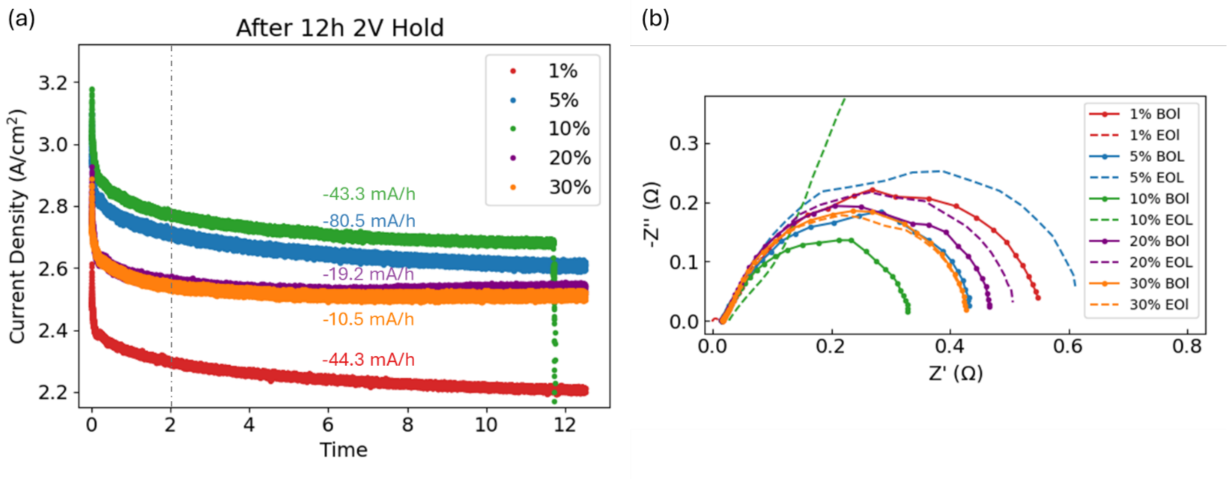

Figure S17. (a) Repeated durability test (2 V hold for 12.5 h) with different powdered ionomer contents. (b) EIS at 1.5 V. EIS with 1% and 10% ionomer contents was not successfully taken at EOL due to cell failure.

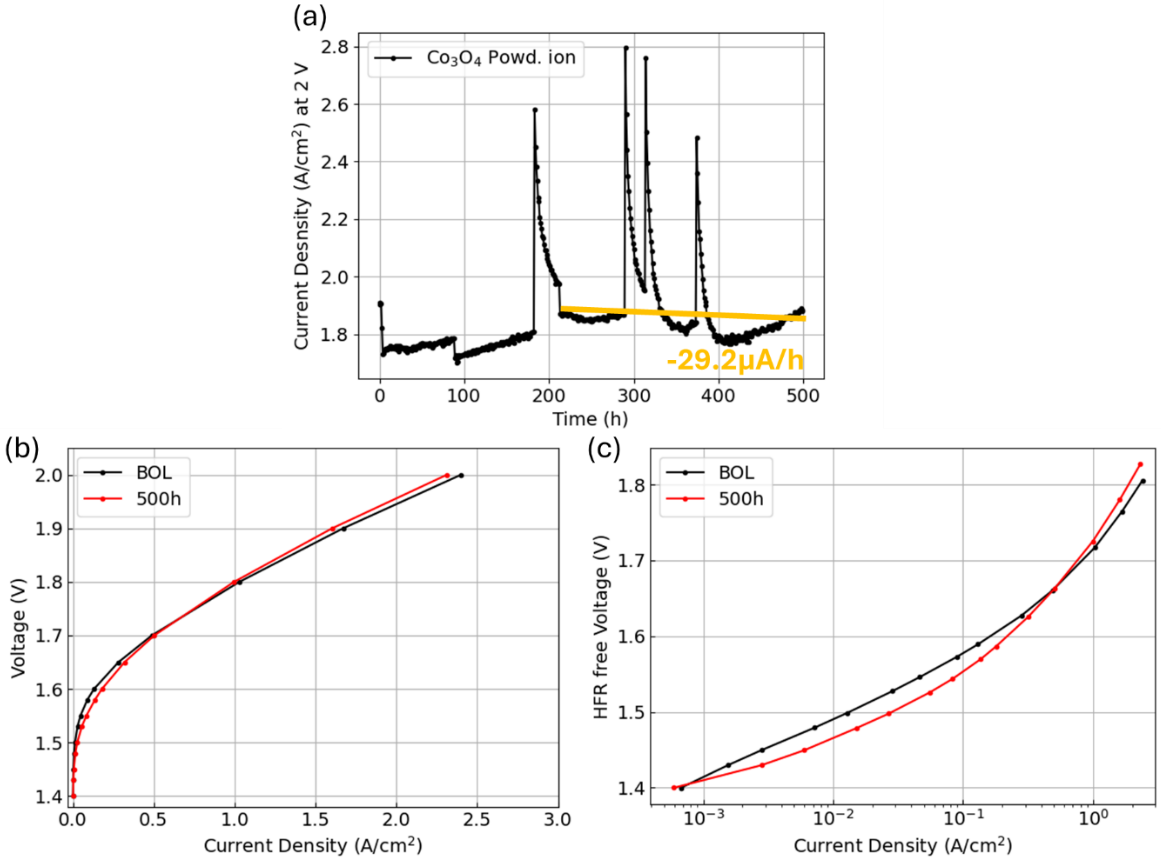

Figure S18. (a) 500 h durability test with Co_3O_4 (0.75 mg_{Co}/cm^2) and 20 wt% of Versogen powdered ionomer at the anode. (b) Polarization curves and (c) HFR-free voltage at BOL and 500 h.

Table S1. The summary of Tafel slope, charge transfer resistance (R_{ct}) and equivalent capacitance (C) obtained by ECM with EIS at 1.5 V and catalyst layer resistance (CLR) from non-faradaic impedance measurement at 1.25 V.

Samples	Tafel slope (mV/dec)	Rct (Ω)	C (F)	CLR (mΩ)
Co_3O_4 Vers Powd.	89.9	0.45	0.51	34.3
Co_3O_4 Vers Disp.	93.7	0.64	0.44	79.4
Co_3O_4 Gen2 Powd.	85.6	0.36	0.45	62.3
Co_3O_4 Gen2 Disp.	94.8	0.46	0.43	82.9
$NiFe_2O_4$ Vers Powd.	80.2	0.33	0.66	72.7
$NiFe_2O_4$ Vers Disp.	85.4	0.41	0.44	149
$NiFe_2O_4$ Gen2 Powd.	79.92	0.23	0.30	68.4
$NiFe_2O_4$ Gen2 Disp.	110	0.21	0.21	80.3
IrO_2 Vers Powd.	85.84	0.38	0.37	85.6
IrO_2 Vers Disp.	96.41	0.46	0.36	130
IrO_2 Gen2 Powd.	85.5	0.24	0.34	73.5
IrO_2 Gen2 Disp.	99.43	0.25	0.38	66.2

Table S2. Co_3O_4 and 1 - 30 wt% Versogen powdered ioomer. The summary of Tafel slope, charge transfer resistance (R_{ct}) and equivalent capacitance (C) obtained by ECM with EIS at 1.5 V and catalyst layer resistance (CLR) from non-faradaic impedance measurement at 1.25 V.

Samples	Tafel slope (mV/dec)	Rct (Ω)	C (F)	CLR (mΩ)
1 %	93.4	0.54	0.69	15.5
5 %	90.1	0.43	0.72	17.5
10 %	85.9	0.37	0.92	18.8
20 %	99.9	0.39	0.80	28.2
30 %	100	0.38	0.68	37.0
